# Frailty Transition and Risk of New-Onset Arthritis Among Adults Aged 45 Years and Older: A Longitudinal Analysis of CHARLS

**DOI:** 10.3390/healthcare14081000

**Published:** 2026-04-10

**Authors:** Yuting Hu, Liangyu Mi, Xinyi Yang, Jinfang Gao, Ke Xu

**Affiliations:** Third Hospital of Shanxi Medical University, Shanxi Bethune Hospital, Shanxi Academy of Medical Sciences, Tongji Shanxi Hospital, Taiyuan 030032, China; hyt15835298892@163.com (Y.H.); mlyidv@hotmail.com (L.M.); yxy9495599@163.com (X.Y.)

**Keywords:** arthritis, frailty transitions, frailty, aging, disease risk, cohort analysis, China Health and Retirement Longitudinal Study

## Abstract

**Highlights:**

**What are the main findings?**

- Worsening frailty over time was associated with a higher risk of incident arthritis in adults aged ≥45 years.
- Frailty improvement was associated with a lower risk of incident arthritis, independent of baseline frailty.

**What are the implications of the main findings?**

- Frailty transitions over time better identifies adults at risk of arthritis than baseline frailty alone.
- Targeting frailty as a modifiable condition may support early prevention strategies and risk reduction for arthritis in aging populations.

**Abstract:**

**Background**: Frailty is a fluctuating health state that may worsen or improve over time and is linked to adverse outcomes, including musculoskeletal disorders such as arthritis. However, evidence on whether frailty changes predict arthritis onset remains limited. This study examined the relationship between changes in frailty status and incident arthritis among Chinese adults aged 45 years and older. **Methods**: We performed a longitudinal cohort analysis using data from the China Health and Retirement Longitudinal Study (CHARLS). Frailty was quantified with a 30-item Frailty Index (FI) and categorized as robust, pre-frail, or frail. Frailty transitions were defined by changes in FI-based categories across survey waves. Incident arthritis was identified as self-reported physician-diagnosed arthritis during follow-up. Associations between frailty transitions and arthritis onset were evaluated using Cox regression, reporting hazard ratios (HRs) and 95% confidence intervals (CIs). Models were adjusted for demographic characteristics, health behaviors, and biochemical indicators, and sensitivity analyses were conducted to verify result stability. **Results**: Among 4982 participants (mean age 58.97 years; 45.58% female). Relative to robust individuals, baseline pre-frailty (HR 1.67, 95% CI 1.41–1.97) and frailty (HR 2.76, 95% CI 1.97–3.85) were associated with higher arthritis risk. Participants whose frailty status worsened from robust to pre-frail or frail also showed higher arthritis risk (HR 1.68, 95% CI 1.34–2.10). In contrast, transitions from frail to pre-frail or robust were associated with lower risk (HR 0.44, 95% CI 0.21–0.92). Higher cumulative frailty burden and greater frailty progression were also associated with increased arthritis risk. **Conclusions**: Frailty transitions are strongly associated with incident self-reported physician-diagnosed arthritis. Monitoring frailty trajectories may improve arthritis risk stratification and support prevention strategies.

## 1. Introduction

Arthritis is a major cause of persistent pain, functional limitation, and reduced well-being in later life [1,2]. In population-based studies, it is typically defined based on self-reported physician diagnosis rather than clinically adjudicated subtypes. As populations age, the health and societal impact of arthritis is increasing worldwide [3,4,5]. According to the 2020 Global Burden of Disease (GBD) study, osteoarthritis (OA) affected approximately 595 million people globally, corresponding to a prevalence of 7.6%, and ranked among the leading causes of years lived with disability in older adults [6,7]. Beyond its substantial health burden, arthritis also imposes considerable economic costs [8,9,10,11,12]. In China, where demographic aging is accelerating, arthritis has become a growing public health concern [13,14,15]. Consequently, identifying modifiable early risk factors has become an urgent priority for disease prevention and management.

Frailty is another aging-related condition that captures multisystem vulnerability and reduced resilience to physiological stress. Even minimal challenges can precipitate adverse events such as falls, disability, hospitalization, or death [16,17], thereby diminishing quality of life and increasing healthcare demands. Importantly, frailty is not a fixed condition but a potentially reversible state [18]. Its progression or improvement can occur naturally or through targeted interventions, including resistance training and nutritional support [19,20]. The concept of “frailty transition” has been shown to provide superior predictive power compared with static frailty measures in several chronic diseases, such as cardiovascular disease [21], chronic kidney disease [22], non-neoplastic gastrointestinal disorders [23], and chronic lung disease [24]. Despite this emerging evidence, how frailty transitions relate to arthritis onset remains insufficiently studied.

Recent research has increasingly examined the association between frailty and arthritis, particularly within the fields of gerontology and musculoskeletal health. Evidence consistently shows that frailty is markedly more prevalent among individuals with arthritis than among those without arthritis [25,26,27], and that frail patients experience worse clinical outcomes [28,29,30]. Prospective cohort studies indicate that baseline frailty predicts future arthritis risk [31], while longitudinal analyses suggest a complex, bidirectional association between the two conditions [32]. Frailty and arthritis may share several broad age-related pathways, including reduced physiological reserve, impaired mobility, metabolic dysfunction, and low-grade systemic inflammation [33,34], although these pathways may differ across arthritis subtypes. Genetic studies using Mendelian randomization further imply overlapping hereditary determinants [35]. Despite these advances, major knowledge gaps persist. Most investigations examine frailty only at baseline or as a static characteristic, overlooking its temporal transitions and their potential impact on arthritis development. Moreover, population-based longitudinal evidence from middle-aged and older adults in China remains limited, hindering the generation of context-specific evidence to inform prevention strategies.

To address these limitations, we used longitudinal data from the China Health and Retirement Longitudinal Study (CHARLS). We examined whether changes in frailty status over time, including both worsening and improvement, were associated with the incidence of arthritis among Chinese adults aged 45 years and older. In the present study, arthritis was assessed as self-reported physician-diagnosed arthritis in a population-based cohort. We hypothesize that worsening frailty would be associated with a higher subsequent risk of incident self-reported physician-diagnosed arthritis, whereas recovery from frailty would be associated with a lower subsequent risk. These findings may support more dynamic approaches to arthritis risk assessment and prevention in both clinical practice and public health settings.

## 2. Materials and Methods

### 2.1. Study Design and Population

**Figure 1:**
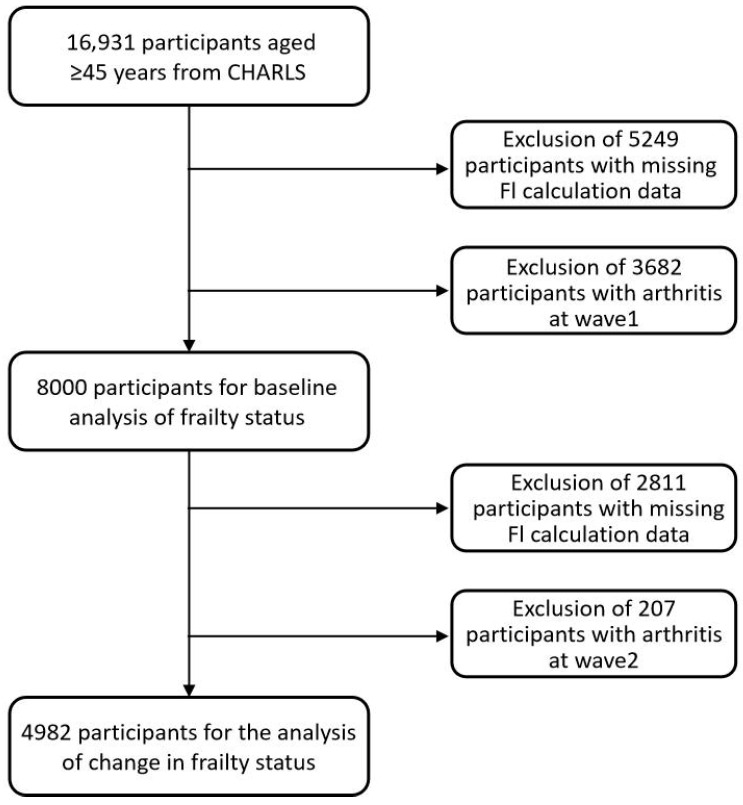
Flowchart depicting study population selection.

The CHARLS is an ongoing longitudinal survey that repeatedly assesses adults aged ≥45 years from 28 provinces in China. It collects extensive data on demographic characteristics, health status, household composition, and socioeconomic conditions [36]. We used Wave 1 (2011) to define baseline characteristics, Wave 2 (2013) to determine frailty transitions, and Waves 3 (2015) and 4 (2018) to capture incident arthritis during follow-up. The CHARLS protocol was reviewed and approved by the Biomedical Ethics Committee of Peking University, and all participants provided written informed consent. Participant selection is outlined in Figure 1. Among 16,931 respondents at baseline, 5249 were excluded because baseline information was insufficient to compute the Frailty Index (FI). After additionally excluding 3682 individuals with baseline self-reported physician-diagnosed arthritis, identified by a “Yes” response to the question “Have you been diagnosed with arthritis by a doctor?” at Wave 1, as well as those with missing follow-up information, 8000 participants remained for baseline analyses. For the transition analysis, additional eligibility criteria were applied at Wave 2 to ensure correct temporal ordering between exposure and outcome. First, 2811 participants with missing data required to compute the FI at Wave 2 were excluded. Subsequently, individuals who reported physician-diagnosed arthritis at Wave 2 or were lost to follow-up were removed (*n* = 207). After these exclusions, 4982 participants who were arthritis-free at both Wave 1 and Wave 2 and had complete frailty measurements were included in the final longitudinal cohort for transition analyses.

Frailty transition was defined using FI values measured at Wave 1 and Wave 2. To ensure that changes in frailty preceded the onset of arthritis and to avoid immortal time bias, we used a landmark design anchored at Wave 2. Follow-up for incident arthritis began after the Wave 2 assessment and continued until the first report of physician-diagnosed arthritis in Wave 3 or 4, death, or the last available interview date, whichever came first.

### 2.2. Assessment of Frailty Status

Frailty status was characterized using a deficit-accumulation Frailty Index (FI), an approach that summarizes overall vulnerability by aggregating multiple age-related health deficits [37,38,39]. Within this framework, frailty is conceptualized as the cumulative burden of deficits across multiple physiological systems, including diseases, symptoms, disabilities, and functional limitations, rather than manifestations of any single disease process. Following established procedures for FI construction, 30 variables were selected encompassing disease conditions (excluding arthritis), physical function, symptom, disability, and cognitive or psychological domains (Appendix A, Table A1). Most items were coded from 0 to 1, with higher values indicating greater deficit severity; the cognition item was treated as a continuous score ranging from 0 to 1. The FI was calculated as the sum of all deficit scores divided by 30, yielding a value from 0 to 1, with higher scores indicating greater frailty. For categorical analyses, participants were grouped into three frailty levels using commonly applied thresholds: robust (FI ≤ 0.10), pre-frail (0.10 < FI < 0.25), and frail (FI ≥ 0.25) [16,40,41]. Frailty transitions were assessed by comparing individual FI values between baseline and the second follow-up. To further capture frailty dynamics, we derived two additional measures: (1) a cumulative frailty burden metric (“total FI”), calculated as the sum of baseline and follow-up FI values, and (2) an FI change score (“FI change”), defined as the difference between follow-up and baseline FI. Both indices were analyzed in tertiles (lowest tertile as the reference) and were also treated as continuous variables to evaluate linear trends.

### 2.3. Covariates

To account for potential confounding, we adjusted for sociodemographic characteristics, lifestyle behaviors, physical activity, and biochemical measures. Sociodemographic variables included sex (male/female), educational attainment (lower than secondary, upper secondary & vocational training, or tertiary education), and marital status (married/partnered vs. other categories, including separated, divorced, unmarried, or widowed). Lifestyle behaviors included smoking (never or ever) and drinking status (never or ever). Physical activity was derived from self-reported frequency and classified into three levels: vigorous activity performed more than once per week, moderate activity performed more than once per week, and inactive (all remaining participants). The continuous covariates were age (years), body mass index (BMI; kg/m^2^), and systolic blood pressure (SBP; mm Hg), triglyceride (TG; mg/dL), C-reactive protein (CRP; mg/L), and glycated hemoglobin (HbA1c; %). BMI, SBP, TG, CRP, and HbA1c were included as baseline covariates in the adjusted models to account for participants’ clinical and metabolic status at study entry. These variables were not modeled as time-varying measures in the present analysis.

### 2.4. Outcome Measurement

The primary outcome of this study was incident self-reported physician-diagnosed arthritis. In each survey wave, participants were asked, “Have you been diagnosed with arthritis by a doctor?” Those who answered “Yes” were considered to have physician-diagnosed arthritis and were asked to report the date of diagnosis, which was used to approximate event onset in the primary analyses. Arthritis subtype information was not available in CHARLS; therefore, the outcome was analyzed as a broad population-based arthritis endpoint rather than as a specific clinical subtype.

Chronic disease diagnoses in CHARLS are repeatedly assessed across survey waves. Participants who report a diagnosis in one wave are asked to confirm it in subsequent waves, and inconsistent reports are retrospectively corrected in the dataset. This repeated verification process may improve the reliability of self-reported physician diagnosis.

Follow-up began in Wave 1 for baseline frailty analyses and in Wave 2 for transition-based analyses. Observation ended at the earliest of arthritis onset, death, or the last available interview date (censoring). The last survey date was considered the review date.

Survival status was determined from the “whether the individual is deceased” variable in Waves 3–4. We estimated the time of death for deceased participants as the total follow-up time minus the baseline follow-up time, as exact dates of death were unavailable. In the main Cox analyses, death was treated as a censoring event, and in the competing-risk analyses, death was treated as a competing event. Because arthritis onset is only observed between survey waves, the event time is subject to interval censoring. Therefore, sensitivity analyses using a wave-based event definition were conducted to assess the robustness of the results.

### 2.5. Statistical Analysis

Baseline characteristics were described using appropriate summary measures. Variables approximating a normal distribution are reported as mean ± standard deviation (SD), while non-normally distributed variables are presented as median with interquartile range (IQR). Categorical data are shown as counts and proportions. Differences across groups were examined using the Kruskal–Wallis H test for continuous variables and the chi-square test for categorical variables.

The association between frailty transitions and incident arthritis was assessed using Cox proportional hazards models, with results presented as hazard ratios (HRs) and 95% confidence intervals (CIs). Schoenfeld residuals confirmed the proportional hazards assumption of these Cox regression models (Appendix A, Table A41). Absolute risk measures, including events, person-years, and incidence rates per 1000 person-years, were also calculated across frailty-transition categories. Person-time was derived in years from interview dates for incidence-rate estimation (Appendix A, Table A42). Robust participants served as the reference category. Covariates were introduced in a stepwise manner: Model 1 included age and sex; Model 2 further adjusted for education level, marital status, smoking, alcohol use, physical activity, BMI, and SBP; Model 3 additionally incorporated HbA1c, CRP, and TG. Missing covariate values (Supplementary Materials, Appendix A, Table A2) were handled using multiple imputation with chained equations (MICE). A total of 20 imputed datasets were generated with 10 iterations each. The imputation model included all variables used in the main regression analyses. The outcome indicator and follow-up time were also included as auxiliary variables in the imputation model, but neither was imputed. Continuous variables were imputed using predictive mean matching, whereas categorical variables were imputed using logistic or multinomial regression models. All variables were analyzed in their original scale without transformation (Appendix A, Table A40). Effect estimates were computed separately for each of the 20 datasets, and then combined according to Rubin’s rules. The multiple imputation was conducted using the R package “mice” in R (version 4.4.0). The imputation focused on missing covariate data used in the regression analyses.

A series of sensitivity analyses were conducted. First, alternative frailty index (FI) cutoffs were used to redefine frailty categories: Cutoff 1—robust FI ≤ 0.10, pre-frail 0.10 < FI ≤ 0.21, frail FI > 0.21 [22]; Cutoff 2—robust FI ≤ 0.08, pre-frail 0.08 < FI < 0.25, frail FI ≥ 0.25 [42]. The main analyses were repeated using these alternative definitions. Second, models were re-estimated with additional adjustments for comorbidities (hypertension, diabetes, heart disease, stroke, and chronic lung disease) and medication use to control for treatment-related confounding. Third, to account for competing risk of death, analyses were repeated using Fine–Gray subdistribution hazards models, with results reported as subdistribution hazard ratios (sHRs) and 95% confidence intervals [43]. Fourth, subgroup analyses were performed by age (<60 vs. ≥60 years) and sex, and interaction was tested using likelihood ratio tests. Fifth, to address uncertainty in the timing of self-reported arthritis diagnosis, we performed a sensitivity analysis using a wave-based event definition, in which incident arthritis was assigned to the survey wave of first report rather than the self-reported diagnosis date. Sixth, to reduce potential overlap between frailty and early musculoskeletal symptoms, we repeated the analyses using a modified 27-item FI after excluding three mobility-related items (difficulty climbing stairs, stooping/kneeling/crouching, and reaching arms overhead). Seventh, to further reduce potential reverse causation, we conducted a sensitivity analysis excluding participants who developed incident arthritis within 2 years after the Wave 2 assessment. Finally, complete-case analyses were conducted as an additional robustness check. All analyses were conducted in R (version 4.4.0). Statistical significance was defined as *p* < 0.05 (two-sided).

## 3. Results

### 3.1. Baseline Characteristics

A total of 8000 participants contributed to the baseline frailty analysis (46.56% female; mean age, 57.72 years), with a median follow-up period of five years. Baseline characteristics are summarized in Table 1. Relative to the robust group, individuals classified as frail differed substantially in both sociodemographic profile and health status. Frail participants were older (mean age, 63.82 years), more frequently female (52.79%), and less likely to be married or partnered (18.27% unmarried). Educational attainment was also lower in the frail group, with only 1.52% reporting college completion. Physiologically, frail individuals exhibited higher systolic blood pressure, lower levels of weekly physical activity, and less favorable biochemical profiles, including elevated triglycerides, C-reactive protein, and HbA1c levels. Participants in the robust group generally had a more favorable health profile, including younger age, fewer deficits, and better metabolic and inflammatory markers, compared with pre-frail and frail participants.

For analyses focused on frailty transitions, 4982 participants met the inclusion criteria (45.58% female; mean age, 58.97 years). Baseline characteristics of these participants, stratified by follow-up frailty status, are summarized in Table 2, and the overall pattern of between-group differences was similar to that observed in the baseline frailty comparison.

A total of 8000 participants were included in the baseline frailty analysis (Table 1), whereas 4982 participants were included in the frailty-transition analysis according to the eligibility criteria shown in Figure 1. The baseline characteristics of the excluded population are shown in Appendix A, Table A39.

### 3.2. Association Between Baseline Frailty Status and Arthritis Risk

Arthritis, defined as self-reported physician-diagnosed arthritis without subtype differentiation, was examined in relation to baseline frailty status. Baseline frailty category was significantly related to subsequent arthritis onset in multivariable-adjusted analyses (Table 3). Using robust participants as the reference group, arthritis risk was higher among those classified as pre-frail (HR = 1.67; 95% CI: 1.41–1.97) and was further elevated among frail individuals (HR = 2.76; 95% CI: 1.97–3.85).

### 3.3. Association Between Transitions in Frailty Status and Arthritis Risk

Table 4 presents baseline frailty status, subsequent transitions, and the corresponding proportion of participants who developed incident arthritis during follow-up. Among the 4982 participants included in the transition analysis, 3663 remained in the same frailty category across the two assessments, 791 experienced progression to a worse state, and 528 showed improvement. Among participants who were robust at baseline, the cumulative incidence of incident arthritis was 17.3% in those who remained robust and 25.7% in those who moved from robust to pre-frail or frail. Conversely, among participants who were frail at baseline, the cumulative incidence of incident arthritis was 43.8%, in those who remained frail and 35.2% in those who improved to pre-frail or robust status. Overall, worsening frailty transition was associated with a higher subsequent risk of incident arthritis, whereas improvement from frailty was associated with a lower subsequent risk.

In addition, absolute risk measures were calculated (Appendix A, Table A42). In absolute terms, arthritis incidence rates generally increased across worsening frailty-transition categories. The lowest rate was observed among participants who remained robust (39.5 per 1000 person-years), whereas substantially higher rates were observed among those who transitioned from robust to pre-frail/frail (63.5 per 1000 person-years), remained pre-frail (79.5 per 1000 person-years), or remained frail (127.0 per 1000 person-years). Overall, these absolute-risk estimates were broadly consistent with the relative-hazard results.

Table 5 summarizes the associations between frailty transitions and the risk of developing arthritis. Relative to individuals who stayed robust, participants who shifted from robust to pre-frail or frail were associated with a higher risk of incident arthritis (HR = 1.68; 95% CI: 1.34–2.10; *p* < 0.001). Within the frail group, improvement to pre-frail or robust status was associated with lower arthritis risk compared with remaining frail (HR = 0.44; 95% CI: 0.21–0.92; *p* = 0.029). Among participants who were pre-frail at baseline, recovery to robustness was linked to reduced arthritis risk (HR = 0.70; 95% CI: 0.52–0.95; *p* = 0.020), although this relationship did not persist after adjustment for demographic factors. Progression from pre-frailty to frailty showed no statistically significant association (HR = 1.15; 95% CI: 0.75–1.76; *p* = 0.518). Consistent with these findings, Table 6 indicates that participants who remained robust across both waves had a lower risk of incident arthritis than those with any history of frailty (HR = 0.53; 95% CI: 0.46–0.62, *p* < 0.001). Overall, progression to frailty was associated with increased arthritis risk, whereas recovery from frailty corresponded to a significant reduction in risk.

### 3.4. Association of Total Frailty Index and Frailty Index Change with Arthritis Risk

Both the total FI and changes in FI were significantly associated with the risk of developing arthritis. This analysis evaluated the influence of overall frailty burden and its longitudinal change across two follow-up periods on arthritis incidence.

Table 7 presents the association between total FI and arthritis risk. Arthritis risk increased across tertiles of total FI. Relative to participants in T1, those in T2 were associated with a higher risk (HR = 1.54; 95% CI: 1.25–1.90; *p* < 0.001), and the association was strongest in T3 (HR = 2.21; 95% CI: 1.81–2.71; *p* < 0.001). A significant positive trend was observed between increasing total FI and arthritis risk (*p* for trend < 0.001), indicating that greater frailty burden was consistently associated with elevated arthritis risk.

Table 8 summarizes the association between FI change (ΔFI) and arthritis risk. Compared with T1, participants in T3 experienced greater arthritis risk (HR = 1.27; 95% CI: 1.07–1.51; *p* = 0.006). In contrast, T2 was associated with lower risk (HR = 0.72; 95% CI: 0.59–0.89; *p* = 0.002), and this pattern persisted after multivariable adjustment. Trend testing suggested that larger ΔFI values were related to higher arthritis risk, reaching statistical significance only in the fully adjusted model (*p* = 0.024). Collectively, these findings indicate that both higher cumulative frailty burden and greater frailty progression are independently related to incident arthritis.

### 3.5. Sensitivity Analysis

A range of additional analyses was performed to evaluate the stability of the main results. First, frailty categories were reconstructed using two alternative FI cut-off schemes (Appendix A, Table A3, Table A4, Table A5, Table A6, Table A7 and Table A8). The direction and magnitude of the associations were unchanged, with frailty worsening remaining associated with higher arthritis risk and frailty improvement remaining associated with lower risk. Building upon Model 3, new models were created by incorporating disease states, medication statuses, and additional covariates to adjust further for potential confounders (Appendix A, Table A9, Table A10, Table A11, Table A12 and Table A13). The results from these models were consistent with the primary analysis. To account for competing risks between mortality and arthritis incidence, a competing risks model was used (Appendix A, Table A14, Table A15, Table A16, Table A17 and Table A18). Competing risk analyses were performed using Fine–Gray subdistribution hazards models, with death treated as a competing event. Subdistribution hazard ratios (sHRs) and 95% confidence intervals were reported. The results from this model were also consistent with the primary findings. We also conducted subgroup analyses stratified by age and sex (Appendix A, Table A19, Table A20, Table A21, Table A22, Table A23, Table A24, Table A25, Table A26, Table A27 and Table A28). Across strata, frailty progression was consistently related to increased arthritis risk. Formal interaction testing showed no statistically significant effect modification by age or sex (interaction *p* > 0.05), indicating that the observed associations were broadly similar across these subgroups. Sensitivity analyses using a wave-based event definition yielded results broadly consistent with the primary analyses (Appendix A, Table A29, Table A30, Table A31, Table A32 and Table A33), indicating that the observed associations were robust to uncertainty in the exact self-reported diagnosis date. Analyses based on the modified 27-item FI also produced similar results (Appendix A, Table A34, Table A35, Table A36, Table A37 and Table A38), suggesting that the main findings were not driven solely by mobility-related components embedded in the original FI. In addition, excluding participants who developed incident arthritis within 2 years after the Wave 2 assessment did not materially change the results (Appendix A, Table A43, Table A44, Table A45, Table A46 and Table A47), which further reduces concern that the observed associations were entirely explained by preclinical or early symptomatic arthritis. Complete-case analyses yielded results that were highly consistent in both direction and magnitude with those obtained from the multiple imputation analyses, with only minor differences in precision (Appendix A, Table A48). Taken together, the sensitivity analyses produced results that mirrored the main analysis.

## 4. Discussion

Using longitudinal data from CHARLS, we examined how baseline frailty status and subsequent frailty trajectories relate to incident self-reported physician-diagnosed arthritis among adults aged 45 years and older. Individuals classified as pre-frail or frail at baseline experienced a higher likelihood of developing arthritis than those who were robust. A similar pattern was observed for frailty transitions: participants whose status shifted toward pre-frailty or frailty showed elevated arthritis risk compared with those who remained robust. In contrast, movement from frailty toward a less frail state was associated with lower arthritis risk relative to persistent frailty. Consistent with these findings, greater cumulative frailty burden (total FI) and larger increases in frailty severity over time (ΔFI) were both linked to higher arthritis incidence.

A growing body of evidence supports the existence of a close link between frailty and arthritis. For example, large population-based studies such as the Korean National Health and Nutrition Examination Survey (KNHANES), the UK Biobank, and the Survey of Health, Aging and Retirement in Europe (SHARE) found that arthritis patients were at increased risk of frailty [26,31,44]. Furthermore, frailty has been associated with higher hospitalization mortality [30] and disease activity among arthritis patients [45]. Evidence from a recent prospective analysis of the National Health and Nutrition Examination Survey (NHANES) further indicates that relatively high frailty indices among US rheumatoid arthritis patients predicted higher all-cause mortality. Moreover, frailty-related mortality increased with age, indicating frailty as a significant risk factor for adverse outcomes in arthritis patients [29]. Despite these findings, most prior research has focused on high-income settings or on selected clinical populations. In contrast, the present study utilized a nationally representative cohort of 4982 middle-aged and older adults from the general Chinese population. Our findings are consistent with earlier cross-sectional and cohort studies, confirming that baseline frail or pre-frail is associated with elevated arthritis risk. Importantly, even after comprehensive adjustment for multiple confounders, frailty was associated with a higher risk of incident self-reported physician-diagnosed arthritis in this population-based cohort, contributing new longitudinal evidence from a Chinese population.

Beyond baseline frailty, we evaluated whether changes in frailty status over time are related to subsequent arthritis onset. To our knowledge, this issue has not been explicitly examined in the existing literature. Frailty is increasingly recognized as a dynamic condition rather than a fixed state. A meta-analysis of community-dwelling older adults reported that after a mean follow-up of 3.9 years, 42.8% of participants transitioned between frailty states. Among those who were pre-frail at baseline, 23.1% transitioned to a robust state, 58.2% remained pre-frail, and 18.2% progressed to frail [46]. Our findings align with this dynamic nature of frailty, extending the understanding of its relationship with arthritis from a static coexistence to a more nuanced understanding of dynamic processes. In this nationally representative Chinese cohort, frailty worsening was consistently associated with higher arthritis incidence. Participants who shifted from robust to pre-frail or frail experienced greater risk than those who remained robust. Additionally, individuals in the pre-frail state who progressed to frailty showed an increased risk of arthritis, though this association did not reach statistical significance in our primary analysis. While our findings are consistent with other studies linking frailty transitions to incident cardiovascular disease [21], they differ from research in other disease areas [22], possibly due to the multifactorial nature of arthritis onset, the nonlinear impact of frailty, or variations in sample size, population characteristics, or arthritis heterogeneity. Notably, sensitivity analyses using different frailty thresholds generally confirmed these results, with some hazard ratios reaching statistical significance, further supporting the clinical relevance of the observed trends. This suggests that the progression from pre-frailty to frailty may contribute to an elevated risk of arthritis. Future studies with larger samples, incorporating objective measures such as joint imaging, are needed for validation. We further quantified frailty dynamics using cumulative frailty burden (total FI) and longitudinal change (ΔFI). Both higher total FI and greater frailty progression were associated with increased arthritis risk, reinforcing the transition-based results. In contrast, frailty recovery was associated with lower subsequent arthritis risk, whether from frail to non-frail or from pre-frail to robust status. This pattern suggests that improvement in frailty may mark a lower-risk trajectory, although causality cannot be inferred from the present observational data. These findings remained robust after adjusting for multiple statistical models and extensive sensitivity analyses. Furthermore, maintaining robustness across both assessments was associated with substantially reduced arthritis risk compared with having pre-frailty or frailty at any time point. Although previous studies have established associations between static frailty status and arthritis incidence and prognosis, for instance, frailty is a predictor of poor outcomes in arthritis patients, particularly in older adults with late-stage arthritis, where frailty is closely linked to pain and osteoporotic fractures [47,48]. Additionally, frailty severity is associated with higher short-term postoperative mortality rates in arthritis patients undergoing joint replacement surgery [49]. However, these findings did not clarify the role of frailty transitions in relation to arthritis risk, a gap that our study fills.

From a biological perspective, the association between frailty and arthritis arises from a complex and interdependent biological network, where frailty dynamics serve as key regulators of the system’s homeostasis. Several broad pathways may help explain the observed association between frailty and incident self-reported physician-diagnosed arthritis, including reduced physiological reserve, impaired physical function, lower physical activity, metabolic dysregulation, and age-related musculoskeletal decline. Low-grade systemic inflammation may also contribute, but this should be interpreted cautiously. Because the outcome in this study was a broad population-based arthritis endpoint without subtype classification, the present findings should not be taken as evidence for a specific inflammatory arthritis mechanism. Frailty progression is commonly accompanied by persistent, low-grade inflammation (“inflammaging”), with higher circulating concentrations of pro-inflammatory mediators such as IL-6 and TNF-α [50,51]. These inflammatory mediators not only accelerate the depletion of multisystem physiological reserves but may also act on joints via the circulatory system, exacerbating synovial inflammation and the senescence-associated secretory phenotype (SASP) of chondrocytes, thereby driving the pathological progression of arthritis [33,52,53,54,55]. In contrast, reducing the inflammatory burden by reversing frailty could create a more protective environment for joint tissues. While low-grade inflammation has been proposed as a potential link between frailty and arthritis, the role of inflammatory pathways should be interpreted with caution in this context. In population-based cohorts, the majority of arthritis cases are likely to represent osteoarthritis, which is primarily driven by mechanical, structural, and age-related factors, although low-grade inflammation may still play a contributory role. Frailty also reflects diminished reserves across multiple organ systems and is often accompanied by reduced muscle strength and physical function [17], leading to altered joint biomechanics and accelerated wear [34,56]. Additionally, frailty is frequently associated with metabolic disorders such as malnutrition [57], vitamin D deficiency [58], and electrolyte imbalances [59,60], all of which contribute to declining musculoskeletal health. In the present study, participants with higher frailty burden had less favorable metabolic profiles, including higher HbA1c and triglyceride levels. Abnormalities in glucose metabolism may contribute to multisystem vulnerability through impaired energy utilization, reduced muscle function, and chronic low-grade inflammation [16,17,51], whereas lipid dysregulation may reflect broader metabolic stress and diminished physiological resilience [16,60]. These metabolic disturbances may jointly increase susceptibility to musculoskeletal disorders and functional decline [33]. Importantly, the association between frailty and incident arthritis remained after adjustment for HbA1c, triglycerides, and CRP, suggesting that the observed relationship was not solely attributable to measured metabolic abnormalities. However, because these variables were included as covariates rather than examined as mediators, their role should be interpreted as a plausible explanatory pathway rather than a confirmed causal mechanism.

Improving muscle strength and restoring physical function during frailty may enhance joint biomechanics, thereby reducing the mechanical stress on the joints. Moreover, the increased burden of comorbidities and genetic factors may influence both frailty and arthritis, highlighting the complexity of the relationship between these conditions [61,62,63]. The transition to frailty reflects these interacting factors, which dynamically modulate the risk of multiple chronic diseases, including arthritis. Thus, the biological mechanism underlying the reduced arthritis risk associated with frailty improvement likely involves reducing systemic inflammation, enhancing physiological function, and stabilizing immune-metabolic homeostasis. This approach not only slows frailty progression but also fosters a more protective intrinsic homeostasis within the joint microenvironment, ultimately delaying the onset and progression of arthritis. This mechanistic framework is consistent with the role of frailty in other chronic diseases, such as chronic lung disease (CLD) and chronic kidney disease (CKD), underscoring the potential of frailty management as a core strategy for preventing multiple chronic conditions.

Our results carry practical implications for both clinical care and population health. Incorporating frailty evaluation into routine midlife and older-adult health assessments—particularly with repeated measurement over time—may improve early identification of individuals at elevated risk for arthritis. Because arthritis is a major contributor to pain-related disability and functional decline, recognizing frailty deterioration before overt joint disease emerges could support earlier preventive action. Importantly, frailty is not necessarily irreversible. The lower arthritis risk observed among participants who moved toward a less frail state suggests that improving frailty may represent a clinically relevant target for future intervention studies and may help identify individuals at potentially lower subsequent risk of arthritis.

Consequently, individuals identified as frail or pre-frail should be prioritized in community-based arthritis prevention initiatives. Early and timely intervention to reverse frailty, through approaches such as nutritional support, resistance training, and physical therapy, is crucial [19,20]. This represents symptomatic treatment to improve current quality of life in older adults. Early intervention targeting frailty may be relevant to prevention-oriented strategies, but whether frailty improvement directly reduces future arthritis risk requires confirmation in interventional studies. Concurrently, frailty management should be regarded as an indispensable component of comprehensive arthritis treatment strategies, particularly during the early stages with a lower comorbidity burden. This calls for collaborative efforts from general practitioners and specialists in orthopedics, rheumatology, and geriatrics to ensure that frailty is properly addressed in arthritis care. Finally, future research should prioritize randomized trials to explore optimal intervention strategies for reversing frailty. Additionally, utilizing multi-omics data to elucidate the underlying mechanisms by which frailty impacts joint health will further provide evidence for precision prevention.

This study has several strengths. First, it evaluates frailty as a time-varying exposure and examines whether frailty worsening or recovery is associated with incident arthritis, extending prior work that has largely relied on baseline frailty measures. Second, the analysis draws on CHARLS, a large nationally representative cohort of community-dwelling Chinese adults, supporting broader applicability of the findings. Third, the results were supported by multiple robustness checks, including adjustments for varying frailty index (FI) thresholds, competing risks models, and multiple comorbidities, which strengthens the reliability and robustness of the results, providing strong evidence for the longitudinal association between frailty dynamics and arthritis onset.

Despite these strengths, several limitations should be considered when interpreting the findings. First, arthritis diagnoses were based on self-reported physician-diagnosed arthritis rather than clinically adjudicated outcomes, and some degree of non-differential misclassification may remain despite repeated verification across survey waves. Moreover, CHARLS does not provide information on arthritis subtypes, so the identified cases in this middle-aged and older cohort likely predominantly reflected osteoarthritis, although this could not be confirmed directly and should therefore be interpreted cautiously. Notably, age-stratified analyses yielded broadly similar results, although this does not resolve the lack of subtype-specific information. In addition, because arthritis onset is only observed between survey waves, event timing is subject to interval censoring. However, sensitivity analyses using a wave-based event definition yielded consistent results, supporting the robustness of our findings. Second, the frailty transition analysis required additional exclusions (*n* = 3018), which may have introduced selection bias. Participants excluded from the transition analysis were generally older and had less favorable baseline health profiles, suggesting that the final analytical sample may represent a relatively healthier subgroup of the original cohort. Therefore, selection bias and reduced representativeness cannot be ruled out, and this may have attenuated or otherwise influenced the observed associations. Nevertheless, multiple sensitivity analyses consistently supported the primary findings. Third, while we adjusted for demographic, behavioral, physiological, and biochemical variables, certain unmeasured confounders, including dietary patterns, vitamin D levels, and genetic factors, could not be accounted for in the analysis. Fourth, the Frailty Index is based on a deficit accumulation framework that incorporates multiple domains of health, including functional and mobility limitations. Some of these components may overlap with early or subclinical manifestations of arthritis, which could introduce exposure contamination and potential reverse causation, possibly leading to overestimation of associations. However, sensitivity analyses using a modified 27-item Frailty Index that excluded mobility-related items produced similar results, suggesting that the observed associations are not driven solely by such overlap. This sensitivity analysis reduces, but does not eliminate, the possibility that early or subclinical musculoskeletal symptoms contributed to frailty measurement before formal arthritis diagnosis. To further minimize potential reverse causation, we conducted a sensitivity analysis excluding participants who developed incident arthritis within 2 years after the Wave 2 assessment. The results were materially unchanged, suggesting that the observed associations are unlikely to be driven by preclinical or undiagnosed arthritis at baseline. Fifth, the CHARLS dataset lacks detailed ICD codes, laboratory metrics, and joint imaging data, precluding classification of arthritis subtypes. Because arthritis represents a heterogeneous group of conditions, including osteoarthritis, rheumatoid arthritis, and gout, the observed associations should be interpreted as applying to incident self-reported physician-diagnosed arthritis as a broad population-based outcome rather than to specific clinical entities. Sixth, our study did not perform component analysis of FI, preventing the identification of specific FI components that most contribute to frailty recovery. Finally, because CHARLS includes adults aged ≥45 years in China, the findings may not generalize to younger populations or to other ethnic and geographic settings. Replication in more diverse cohorts and with clinically adjudicated arthritis outcomes is warranted.

## 5. Conclusions

Using longitudinal data from CHARLS, we found that frailty trajectories are closely related to incident self-reported physician-diagnosed arthritis among Chinese adults aged ≥45 years. Worsening frailty status over time was associated with higher arthritis risk, whereas improvement toward a less frail state was associated with lower risk. These results support the clinical value of incorporating frailty assessment—particularly repeated, dynamic evaluation—into routine health screening and risk stratification for middle-aged and older adults. Interventions that prevent frailty deterioration or promote frailty recovery may represent a practical approach to reducing future arthritis burden, highlighting the importance of early identification and ongoing monitoring in populations at risk.

## Figures and Tables

**Table 1:** Participant characteristics at baseline, stratified by baseline frailty category.

Characteristic	Overall	Frailty Status			*p* Value
		Robust	Pre-Frail	Frail	
*n*	8000	5504	2102	394	
Age, mean (SD), years	57.72 (9.09)	56.41 (8.57)	59.99 (9.42)	63.82 (9.49)	<0.001
Sex, *n* (%)					<0.001
Male	4275 (53.44%)	3120 (56.69%)	969 (46.10%)	186 (47.21%)	
Female	3725 (46.56%)	2384 (43.31%)	1133 (53.90%)	208 (52.79%)	
Smoking status, *n* (%)					0.024
Never smokers	4574 (57.18%)	3093 (56.21%)	1254 (59.66%)	227 (57.61%)	
Ever smokers	3425 (42.82%)	2410 (43.79%)	848 (40.34%)	167 (42.39%)	
Drinking status, *n* (%)					<0.001
Never drinkers	4648 (58.11%)	3096 (56.26%)	1316 (62.61%)	236 (59.90%)	
Ever drinkers	3351 (41.89%)	2407 (43.74%)	786 (37.39%)	158 (40.10%)	
Marital status, *n* (%)					<0.001
Other marital status	816 (10.20%)	462 (8.39%)	282 (13.42%)	72 (18.27%)	
Married or partnered	7184 (89.80%)	5042 (91.61%)	1820 (86.58%)	322 (81.73%)	
Education, *n* (%)					<0.001
Lower than secondary	6720 (84.00%)	4477 (81.34%)	1876 (89.25%)	367 (93.15%)	
Upper secondary & vocational training	1061 (13.26%)	858 (15.59%)	182 (8.66%)	21 (5.33%)	
Tertiary education	219 (2.74%)	169 (3.07%)	44 (2.09%)	6 (1.52%)	
Physical activity, *n* (%)					<0.001
Light	2633 (41.18%)	1729 (38.60%)	741 (45.21%)	163 (59.06%)	
Moderate	1882 (29.43%)	1337 (29.85%)	475 (28.98%)	70 (25.36%)	
Vigorous	1879 (29.39%)	1413 (31.55%)	423 (25.81%)	43 (15.58%)	
BMI, median (IQR), kg/m^2^	23.19 (20.93–25.64)	23.13 (20.97–25.46)	23.38 (20.86–26.03)	23.13 (20.77–26.15)	0.200
SBP, mean (SD), mmHg	129.08 (20.97)	127.44 (19.93)	131.97 (22.19)	135.74 (24.58)	<0.001
HbA1c, mean (SD), %	5.25 (0.82)	5.20 (0.72)	5.34 (0.95)	5.48 (1.16)	<0.001
TG, mean (SD), mg/dL	133.11 (106.34)	129.50 (100.37)	140.37 (117.88)	141.30 (114.48)	<0.001
CRP, median (IQR), mg/L	1.02 (0.54–2.09)	0.95 (0.52–1.95)	1.14 (0.58–2.38)	1.18 (0.63–2.50)	<0.001

Continuous variables were compared across groups using the Kruskal–Wallis H test, and categorical variables using the chi-square test. BMI, body mass index; SBP, systolic blood pressure; HbA1c, glycated hemoglobin; TG, triglycerides; CRP, C-reactive protein; IQR, interquartile range.

**Table 2:** Baseline participant characteristics according to follow-up frailty status.

Characteristic	Overall	Frailty Status			*p* Value
		Robust	Pre-Frail	Frail	
*n*	4982	3472	1261	249	
Age, mean (SD), years	58.97 (8.54)	58.00 (8.20)	60.78 (8.76)	63.39 (9.16)	<0.001
Sex, *n* (%)					<0.001
Male	2711 (54.42%)	2008 (57.83%)	577 (45.76%)	126 (50.60%)	
Female	2271 (45.58%)	1464 (42.17%)	684 (54.24%)	123 (49.40%)	
Smoking status, *n* (%)					0.003
Never smokers	2672 (53.63%)	1810 (52.13%)	727 (57.65%)	135 (54.22%)	
Ever smokers	2310 (46.37%)	1662 (47.87%)	534 (42.35%)	114 (45.78%)	
Drinking status, *n* (%)					<0.001
Never drinkers	2536 (51.00%)	1693 (48.80%)	712 (56.73%)	131 (52.61%)	
Ever drinkers	2437 (49.00%)	1776 (51.20%)	543 (43.27%)	118 (47.39%)	
Marital status, *n* (%)					<0.001
Other marital status	473 (9.49%)	287 (8.27%)	142 (11.26%)	44 (17.67%)	
Married or partnered	4509 (90.51%)	3185 (91.73%)	1119 (88.74%)	205 (82.33%)	
Education, *n* (%)					<0.001
Lower than secondary	4150 (83.30%)	2822 (81.28%)	1107 (87.79%)	221 (88.76%)	
Upper secondary & vocational training	715 (14.35%)	559 (16.10%)	134 (10.63%)	22 (8.84%)	
Tertiary education	117 (2.35%)	91 (2.62%)	20 (1.59%)	6 (2.41%)	
Physical activity, *n* (%)					<0.001
Light	1682 (39.35%)	1120 (36.95%)	460 (43.31%)	102 (56.04%)	
Moderate	1321 (30.90%)	938 (30.95%)	338 (31.83%)	45 (24.73%)	
Vigorous	1272 (29.75%)	973 (32.10%)	264 (24.86%)	35 (19.23%)	
BMI, median (IQR), kg/m^2^	23.65 (21.30–26.17)	23.54 (21.30–25.97)	23.99 (21.31–26.52)	23.76 (21.32–26.58)	0.029
SBP, mean (SD), mmHg	128.79 (20.54)	127.73 (19.89)	130.14 (20.93)	136.43 (25.06)	<0.001
HbA1c, mean (SD), %	5.25 (0.80)	5.18 (0.69)	5.35 (0.94)	5.61 (1.17)	<0.001
TG, mean (SD), mg/dL	133.49 (103.07)	130.97 (106.28)	137.82 (95.51)	144.99 (95.43)	<0.001
CRP, median (IQR), mg/L	1.02 (0.54–2.08)	0.95 (0.52–1.92)	1.16 (0.59–2.37)	1.33 (0.72–2.96)	<0.001

Group differences were assessed with the Kruskal–Wallis H test (continuous variables) and the chi-square test (categorical variables). BMI, body mass index; SBP, systolic blood pressure; HbA1c, glycated hemoglobin; TG, triglycerides; CRP, C-reactive protein; IQR, interquartile range.

**Table 3:** Baseline frailty category and incident self-reported physician-diagnosed arthritis.

Frailty Status	Events/*n*	Model 1			Model 2			Model 3		
		HR	95% CI	*p*	HR	95% CI	*p*	HR	95% CI	*p*
Robust	691/3680	1.00	reference	-	1.00	reference	-	1.00	reference	-
Pre-frail	342/1147	1.70	1.49–1.94	<0.001	1.71	1.48–1.98	<0.001	1.67	1.41–1.97	<0.001
Frail	60/155	2.35	1.80–3.06	<0.001	2.84	2.11–3.83	<0.001	2.76	1.97–3.85	<0.001

Model 1: age, sex. Model 2: Model 1 + education, marital status, smoking, drinking, physical activity, BMI, SBP. Model 3: Model 2 + HbA1c, CRP, TG. HR, hazard ratio; CI: confidence interval.

**Table 4:** Number and percentage of participants by frailty status and new-onset arthritis cases.

Baseline	New-Onset Arthritis Patients; N (%)	The Second Survey	Changes in Frailty Status; N (%)	New-Onset Arthritis Patients; N (%)
Robust	691 (18.8%)	Robust	3018 (82.0%)	521 (17.3%)
		Pre-frail/Frail	662 (18.0%)	170 (25.7%)
Pre-frail	342 (29.8%)	Robust	437 (38.1%)	119 (27.2%)
		Pre-frail	581 (50.7%)	179 (30.8%)
		Frail	129 (11.2%)	44 (34.1%)
Frail	60 (38.7%)	Robust/Pre-frail	91 (58.7%)	32 (35.2%)
		Frail	64 (41.3%)	28 (43.8%)

The interval between baseline and the second CHARLS assessment was two years.

**Table 5:** Incident self-reported physician-diagnosed arthritis risk by frailty transition group.

Frailty Transition	Events/*n*	Model 1			Model 2			Model 3		
		HR	95% CI	*p*	HR	95% CI	*p*	HR	95% CI	*p*
Stable robust	521/3018	1.00	reference	-	1.00	reference	-	1.00	reference	-
Robust to pre-frail/frail	170/662	1.59	1.33–1.89	<0.001	1.69	1.40–2.05	<0.001	1.68	1.34–2.10	<0.001
Stable pre-frail	179/581	1.00	reference	-	1.00	reference	-	1.00	reference	-
Pre-frail to robust	119/437	0.86	0.68–1.08	0.199	0.84	0.65–1.09	0.185	0.70	0.52–0.95	0.020
Pre-frail to frail	44/129	1.23	0.88–1.71	0.226	1.14	0.77–1.68	0.508	1.15	0.75–1.76	0.518
Stable frail	28/64	1.00	reference	-	1.00	reference	-	1.00	reference	-
Frail to robust/pre-frail	32/91	0.84	0.49–1.41	0.502	0.49	0.26–0.93	0.030	0.44	0.21–0.92	0.029

Model 1: age, sex. Model 2: Model 1 + education, marital status, smoking, drinking, physical activity, BMI, SBP. Model 3: Model 2 + HbA1c, CRP, TG. HR, hazard ratio; CI: confidence interval.

**Table 6:** Arthritis risk according to frailty status across two assessments.

Frailty Status of Two Surveys	Events/*n*	Model 1			Model 2			Model 3		
		HR	95% CI	*p*	HR	95% CI	*p*	HR	95% CI	*p*
Frail or pre-frail	572/1964	1.00	reference	-	1.00	reference	-	1.00	reference	-
Stable robust	521/3018	0.55	0.49–0.62	<0.001	0.53	0.46–0.62	<0.001	0.53	0.46–0.62	<0.001

Model 1: age, sex. Model 2: Model 1 + education, marital status, smoking, drinking, physical activity, BMI, SBP. Model 3: Model 2 + HbA1c, CRP, TG. HR, hazard ratio; CI: confidence interval.

**Table 7:** Total FI tertiles and incident self-reported physician-diagnosed arthritis risk.

Term	Events/*n*	Model 1			Model 2			Model 3		
		HR	95% CI	*p*	HR	95% CI	*p*	HR	95% CI	*p*
T1 of total FI	249/1649	1.00	reference	-	1.00	reference	-	1.00	reference	-
T2 of total FI	358/1643	1.49	1.26–1.75	<0.001	1.59	1.33–1.90	<0.001	1.54	1.25–1.90	<0.001
T3 of total FI	486/1690	2.08	1.78–2.43	<0.001	2.33	1.96–2.77	<0.001	2.21	1.81–2.71	<0.001
*p* for trend				<0.001			<0.001			<0.001

Total FI = FI (baseline) + FI (second survey). Participants were grouped into tertiles (T1–T3). Model 1: age, sex. Model 2: Model 1 + education, marital status, smoking, drinking, physical activity, BMI, SBP. Model 3: Model 2 + HbA1c, CRP, TG. HR, hazard ratio; CI: confidence interval.

**Table 8:** ΔFI tertiles and incident self-reported physician-diagnosed arthritis risk.

Term	Events/*n*	Model 1			Model 2			Model 3		
		HR	95% CI	*p*	HR	95% CI	*p*	HR	95% CI	*p*
T1 of ΔFI	397/1692	1.00	reference	-	1.00	reference	-	1.00	reference	-
T2 of ΔFI	262/1596	0.68	0.58–0.79	<0.001	0.72	0.61–0.85	<0.001	0.72	0.59–0.89	0.002
T3 of ΔFI	434/1694	1.09	0.95–1.25	0.218	1.19	1.02–1.38	0.023	1.27	1.07–1.51	0.006
*p* for trend				0.689			0.228			0.024

ΔFI = FI (second survey) − FI (baseline). Model 1: age, sex. Model 2: Model 1 + education, marital status, smoking, drinking, physical activity, BMI, SBP. Model 3: Model 2 + HbA1c, CRP, TG. HR, hazard ratio; CI: confidence interval.

## Data Availability

The datasets used in this study are publicly available on the China Health and Retirement Longitudinal Study (CHARLS) website at https://charls.pku.edu.cn/ (31 July 2025). Data generated and analyzed during this study are available from the corresponding author upon reasonable request.

## Supplementary Materials

The following supporting information can be downloaded at https://www.mdpi.com/article/10.3390/healthcare14081000/s1. References [64,65,66] are cited in the Supplementary Materials.

## Abbreviations

The following abbreviations are used in this manuscript:

CHARLS	China Health and Retirement Longitudinal Study
FI	Frailty Index
HR	Hazard Ratio
CI	Confidence Interval
OA	Osteoarthritis
RA	Rheumatoid Arthritis
GBD	Global Burden of Disease
YLD	Years Lived with Disability
BMI	Body Mass Index
SBP	Systolic Blood Pressure
TG	Triglyceride
CRP	C-reactive protein
HbA1c	Glycated hemoglobin
SD	Standard Deviation
IQR	Interquartile Range
CLD	Chronic Lung Disease
CKD	Chronic Kidney Disease

## Appendix A

**Table A1:** The 30 items used to construct the frailty index.

Number	Description of the Item	Cut-off Value
1	Self-reported physician diagnosed hypertension	Yes = 1, No = 0
2	Self-reported physician diagnosed diabetes	Yes = 1, No = 0
3	Self-reported physician diagnosed cancer	Yes = 1, No = 0
4	Self-reported physician diagnosed chronic lung disease	Yes = 1, No = 0
5	Self-reported physician diagnosed any emotional, nervous, or psychiatric problems	Yes = 1, No = 0
6	Self-reported physician diagnosed memory-related disease	Yes = 1, No = 0
7	Self-reported physician diagnosed heart problem	Yes = 1, No = 0
8	Self-reported physician diagnosed stroke	Yes = 1, No = 0
9	Self-reported vision problems	Yes = 1, No = 0
10	Self-reported hearing problems	Yes = 1, No = 0
11	Self-reported general health status	Very poor or poor = 1, Very good, good, or fair = 0
12	Difficulty with dressing	Yes = 1, No = 0
13	Difficulty with bathing or showering	Yes = 1, No = 0
14	Difficulty with eating	Yes = 1, No = 0
15	Difficulty with getting in and out of bed	Yes = 1, No = 0
16	Difficulty with using the toilet	Yes = 1, No = 0
17	Difficulty with managing money	Yes = 1, No = 0
18	Difficulty with taking medication	Yes = 1, No = 0
19	Difficulty with shopping for groceries	Yes = 1, No = 0
20	Difficulty with controlling urination and defecation	Yes = 1, No = 0
21	Difficulty with preparing meals	Yes = 1, No = 0
22	Mobility: difficulty with walking 100 yards or one block	Yes = 1, No = 0
23	Mobility: difficulty with getting up from a chair after sitting for long periods	Yes = 1, No = 0
24	Mobility: difficulty with climbing several flights of stairs without resting	Yes = 1, No = 0
25	Mobility: difficulty with lifting or carrying weights over 10 pounds/jins	Yes = 1, No = 0
26	Mobility: difficulty with picking up a coin from the table	Yes = 1, No = 0
27	Mobility: difficulty with stooping, kneeling, or crouching	Yes = 1, No = 0
28	Mobility: difficulty with reaching arms above shoulder level	Yes = 1, No = 0
29	Depression: CESD-10 questionnaire	CESD-10 >10 = 1, ≤10 = 0
30	Cognition: (memory test score + orientation test score)/14	Continuous variable, ranging from 0 to 1

Memory-related diseases were defined as Alzheimer’s disease, dementia, organic brain senility, and other severe memory impairment. Depressive symptoms were measured in CHARLS using the 10-item Center for Epidemiologic Studies Depression Scale (CESD-10), producing a total score from 0 to 30. The memory score (0–10) was derived from performance on immediate and delayed word-recall tasks and was calculated as the mean number of words not recalled across the two tests. Temporal orientation was evaluated using four items (day of the week, month, date, and year); the orientation score ranged from 0 to 4, with one point assigned for each incorrect answer.

**Table A2:** Missingness summary for covariates.

Variable	Baseline Frailty Status		Frailty Status Changes	
	Missing Number	Missing Proportion (%)	Missing Number	Missing Proportion (%)
Age	0	0.00	0	0.00
Gender	0	0.00	0	0.00
Educational level	0	0.00	0	0.00
Marital status	0	0.00	0	0.00
Alcohol consumption	1	0.01	1	0.02
BMI	1214	15.17	683	13.71
SBP	1226	15.32	690	13.85
Smoking status	1	0.01	1	0.02
Physical activity	0	0.00	0	0.00
TG	2562	32.02	1390	27.90
CRP	2560	32.00	1388	27.86
HbA1c	2534	31.67	1363	27.36

BMI, body mass index; SBP, systolic blood pressure; TG, triglycerides; CRP, C-reactive protein; HbA1c, glycated hemoglobin.

**Table A3:** Baseline frailty category and incident arthritis risk using FI cut-off 1.

Frailty Status	Events/*n*	Model 1			Model 2			Model 3		
		HR	95% CI	*p*	HR	95% CI	*p*	HR	95% CI	*p*
Robust	691/3680	1.00 (reference)	–	–	1.00 (reference)	–	–	1.00 (reference)	–	–
Pre-frail	296/1016	1.65	1.43–1.89	<0.001	1.66	1.43–1.93	<0.001	1.63	1.37–1.94	<0.001
Frail	106/286	2.24	1.82–2.76	<0.001	2.49	1.98–3.15	<0.001	2.39	1.83–3.11	<0.001

FI cut-off 1 definition: robust (FI ≤ 0.10); pre-frail (0.10 < FI ≤ 0.21); frail (FI > 0.21). Model 1: age, sex. Model 2: Model 1 + education, marital status, smoking, drinking, physical activity, BMI, SBP. Model 3: Model 2 + HbA1c, CRP, TG. HR, hazard ratio; CI: confidence interval.

**Table A4:** Frailty transition group and incident arthritis risk using FI cut-off 1.

Frailty Transition	Events/*n*	Model 1			Model 2			Model 3		
		HR	95% CI	*p*	HR	95% CI	*p*	HR	95% CI	*p*
Stable robust	521/3018	1.00 (reference)	–	–	1.00 (reference)	–	–	1.00 (reference)	–	–
Robust to pre-frail/frail	170/662	1.59	1.33–1.89	<0.001	1.69	1.40–2.05	<0.001	1.68	1.34–2.10	<0.001
Stable pre-frail	127/424	1.00 (reference)	–	–	1.00 (reference)	–	–	1.00 (reference)	–	–
Pre-frail to robust	105/407	0.83	0.64–1.08	0.158	0.83	0.62–1.10	0.200	0.71	0.51–0.99	0.044
Pre-frail to frail	64/185	1.26	0.93–1.70	0.138	1.25	0.89–1.75	0.199	1.34	0.92–1.94	0.129
Stable frail	58/137	1.00 (reference)	–	–	1.00 (reference)	–	–	1.00 (reference)	–	–
Frail to robust/pre-frail	48/149	0.76	0.51–1.12	0.163	0.55	0.35–0.87	0.01	0.49	0.29–0.82	0.007

FI cut-off 1 definition: robust (FI ≤ 0.10); pre-frail (0.10 < FI ≤ 0.21); frail (FI > 0.21). Model 1: age, sex. Model 2: Model 1 + education, marital status, smoking, drinking, physical activity, BMI, SBP. Model 3: Model 2 + HbA1c, CRP, TG. HR, hazard ratio; CI: confidence interval.

**Table A5:** Frailty status across two assessments and incident arthritis risk using FI cut-off 1.

Frailty Status of Two Survey	Events/*n*	Model 1			Model 2			Model 3		
		HR	95% CI	*p*	HR	95% CI	*p*	HR	95% CI	*p*
Frail or pre-frail	572/1964	1.00 (reference)	–	–	1.00 (reference)	–	–	1.00 (reference)	–	–
Stable robust	521/3018	0.55	0.49–0.62	<0.001	0.53	0.46–0.60	<0.001	0.53	0.46–0.62	<0.001

FI cut-off 1 definition: robust (FI ≤ 0.10); pre-frail (0.10 < FI ≤ 0.21); frail (FI > 0.21). Model 1: age, sex. Model 2: Model 1 + education, marital status, smoking, drinking, physical activity, BMI, SBP. Model 3: Model 2 + HbA1c, CRP, TG. HR, hazard ratio; CI: confidence interval.

**Table A6:** Baseline frailty category and incident arthritis risk using FI cut-off 2.

Frailty Status	Events/*n*	Model 1			Model 2			Model 3		
		HR	95% CI	*p*	HR	95% CI	*p*	HR	95% CI	*p*
Robust	559/3096	1.00 (reference)	–	–	1.00 (reference)	–	–	1.00 (reference)	–	–
Pre-frail	474/1731	1.60	1.41–1.80	<0.001	1.64	1.43–1.88	<0.001	1.53	1.31–1.79	<0.001
Frail	60/155	2.44	1.87–3.20	<0.001	2.98	2.20–4.03	<0.001	2.82	2.01–3.96	<0.001

FI cut-off 2 definition: robust (FI ≤ 0.08); pre-frail (0.08 < FI < 0.25); frail (FI ≥ 0.25). Model 1: age, sex. Model 2: Model 1 + education, marital status, smoking, drinking, physical activity, BMI, SBP. Model 3: Model 2 + HbA1c, CRP, TG. HR, hazard ratio; CI: confidence interval.

**Table A7:** Frailty transition group and incident arthritis risk using FI cut-off 2.

Frailty Transition	Events/*n*	Model 1			Model 2			Model 3		
		HR	95% CI	*p*	HR	95% CI	*p*	HR	95% CI	*p*
Stable robust	359/2312	1.00 (reference)	–	–	1.00 (reference)	–	–	1.00 (reference)	–	–
Robust to pre-frail/frail	200/784	1.76	1.48–2.10	<0.001	1.92	1.58–2.33	<0.001	1.88	1.50–2.35	<0.001
Stable pre-frail	265/1032	1.00 (reference)	–	–	1.00 (reference)	–	–	1.00 (reference)	–	–
Pre-frail to robust	159/553	1.14	0.93–1.39	0.197	1.07	0.86–1.32	0.567	1.03	0.80–1.33	0.809
Pre-frail to frail	50/146	1.52	1.13–2.07	0.006	1.44	1.01–2.05	0.042	1.62	1.10–2.39	0.016
Stable frail	28/64	1.00 (reference)	–	–	1.00 (reference)	–	–	1.00 (reference)	–	–
Frail to robust/pre-frail	32/91	0.84	0.49–1.41	0.502	0.49	0.26–0.93	0.030	0.44	0.21–0.92	0.029

FI cut-off 2 definition: robust (FI ≤ 0.08); pre-frail (0.08 < FI < 0.25); frail (FI ≥ 0.25). Model 1: age, sex. Model 2: Model 1 + education, marital status, smoking, drinking, physical activity, BMI, SBP. Model 3: Model 2 + HbA1c, CRP, TG. HR, hazard ratio; CI: confidence interval.

**Table A8:** Frailty status across two assessments and incident arthritis risk using FI cut-off 2.

Frailty Status of Two Survey	Events/*n*	Model 1			Model 2			Model 3		
		HR	95% CI	*p*	HR	95% CI	*p*	HR	95% CI	*p*
Frail or pre-frail	734/2670	1.00 (reference)	–	–	1.00 (reference)	–	–	1.00 (reference)	–	–
Stable robust	359/2312	0.53	0.46–0.60	<0.001	0.50	0.43–0.57	<0.001	0.52	0.44–0.61	<0.001

FI cut-off 2 definition: robust (FI ≤ 0.08); pre-frail (0.08 < FI < 0.25); frail (FI ≥ 0.25). Model 1: age, sex. Model 2: Model 1 + education, marital status, smoking, drinking, physical activity, BMI, SBP. Model 3: Model 2 + HbA1c, CRP, TG. HR, hazard ratio; CI: confidence interval.

**Table A9:** Baseline frailty category and incident arthritis risk with additional adjustment for multimorbidity and medication status.

Frailty Status	Events/*n*	Model 1			Model 2			Model 3		
		HR	95% CI	*p*	HR	95% CI	*p*	HR	95% CI	*p*
Robust	691/3680	1.00 (reference)	–	–	1.00 (reference)	–	–	1.00 (reference)	–	–
Pre-frail	342/1147	1.85	1.55–2.21	<0.001	1.73	1.45–2.06	<0.001	1.85	1.54–2.21	<0.001
Frail	60/155	3.41	2.38–4.88	<0.001	2.94	2.05–4.22	<0.001	3.32	2.30–4.80	<0.001

Main covariates: age, sex, education level, marital status, smoking status, drinking status, physical activity, BMI, SBP, HbA1c, CRP, TG. Model 1: main covariates + multimorbidity state. Model 2: main covariates + medication status. Model 3: main covariates + multimorbidity state + medication status.

**Table A10:** Frailty transition group and incident arthritis risk with additional adjustment for multimorbidity and medication status.

Frailty Transition	Events/*n*	Model 1			Model 2			Model 3		
		HR	95% CI	*p*	HR	95% CI	*p*	HR	95% CI	*p*
Stable robust	521/3018	1.00 (reference)	–	–	1.00 (reference)	–	–	1.00 (reference)	–	–
Robust to pre-frail/frail	170/662	1.80	1.43–2.27	<0.001	1.67	1.33–2.09	<0.001	1.81	1.43–2.28	<0.001
Stable pre-frail	179/581	1.00 (reference)	–	–	1.00 (reference)	–	–	1.00 (reference)	–	–
Pre-frail to robust	119/437	0.60	0.44–0.82	0.001	0.63	0.46–0.86	0.003	0.59	0.43–0.80	<0.001
Pre-frail to frail	44/129	1.15	0.75–1.76	0.511	1.18	0.77–1.80	0.451	1.18	0.77–1.80	0.451
Stable frail	28/64	1.00 (reference)	–	–	1.00 (reference)	–	–	1.00 (reference)	–	–
Frail to robust/pre-frail	32/91	0.36	0.14–0.95	0.038	0.44	0.19–1.01	0.053	0.32	0.12–0.84	0.021

Main covariates: age, sex, education level, marital status, smoking status, drinking status, physical activity, BMI, SBP, HbA1c, CRP, TG. Model 1: main covariates + multimorbidity state. Model 2: main covariates + medication status. Model 3: main covariates + multimorbidity state + medication status.

**Table A11:** Frailty status across two assessments and incident arthritis risk with additional adjustment for multimorbidity and medication status.

Frailty Status of Two Survey	Events/*n*	Model 1			Model 2			Model 3		
		HR	95% CI	*p*	HR	95% CI	*p*	HR	95% CI	*p*
Frail or pre-frail	572/1964	1.00 (reference)	–	–	1.00 (reference)	–	–	1.00 (reference)	–	–
Stable robust	521/3018	0.49	0.41–0.57	<0.001	0.52	0.45–0.62	<0.001	0.49	0.41–0.58	<0.001

Main covariates: age, sex, education level, marital status, smoking status, drinking status, physical activity, BMI, SBP, HbA1c, CRP, TG. Model 1: main covariates + multimorbidity state. Model 2: main covariates + medication status. Model 3: main covariates + multimorbidity state + medication status.

**Table A12:** Total FI and incident arthritis risk with additional adjustment for multimorbidity and medication status.

Term	Events/*n*	Model 1			Model 2			Model 3		
		HR	95% CI	*p*	HR	95% CI	*p*	HR	95% CI	*p*
T1 of total FI	249/1649	1.00 (reference)	–	–	1.00 (reference)	–	–	1.00 (reference)	–	–
T2 of total FI	358/1643	1.68	1.36–2.08	<0.001	1.56	1.27–1.93	<0.001	1.68	1.36–2.08	<0.001
T3 of total FI	486/1690	2.73	2.18–3.41	<0.001	2.32	1.87–2.88	<0.001	2.71	2.17–3.40	<0.001
*p* for trend		<0.001			<0.001			<0.001		

Main covariates: age, sex, education level, marital status, smoking status, drinking status, physical activity, BMI, SBP, HbA1c, CRP, TG. Model 1: main covariates + multimorbidity state. Model 2: main covariates + medication status. Model 3: main covariates + multimorbidity state + medication status.

**Table A13:** ΔFI and incident arthritis risk with additional adjustment for multimorbidity and medication status.

Term	Events/*n*	Model 1			Model 2			Model 3		
		HR	95% CI	*p*	HR	95% CI	*p*	HR	95% CI	*p*
T1 of ΔFI	397/1692	1.00 (reference)	–	–	1.00 (reference)	–	–	1.00 (reference)	–	–
T2 of ΔFI	262/1596	0.72	0.59–0.89	0.002	0.73	0.60–0.89	0.002	0.72	0.59–0.88	0.001
T3 of ΔFI	434/1694	1.27	1.07–1.51	0.006	1.27	1.07–1.51	0.007	1.28	1.07–1.52	0.006
*p* for trend		0.023			0.020			0.018		

Main covariates: age, sex, education level, marital status, smoking status, drinking status, physical activity, BMI, SBP, HbA1c, CRP, TG. Model 1: main covariates + multimorbidity state. Model 2: main covariates + medication status. Model 3: main covariates + multimorbidity state + medication status.

**Table A14:** Baseline frailty category and incident arthritis risk in Fine–Gray competing-risk analysis.

Frailty Status	Events/*n*	Model 1			Model 2			Model 3		
		sHR	95% CI	*p*	sHR	95% CI	*p*	sHR	95% CI	*p*
Robust	691/3680	1.00 (reference)	–	–	1.00 (reference)	–	–	1.00 (reference)	–	–
Pre-frail	342/1147	1.67	1.47–1.90	<0.001	1.69	1.46–1.94	<0.001	1.64	1.39–1.93	<0.001
Frail	60/155	2.31	1.78–3.00	<0.001	2.77	2.07–3.72	<0.001	2.65	1.91–3.67	<0.001

Competing event: mortality. Model 1: age, sex. Model 2: Model 1 + education, marital status, smoking, drinking, physical activity, BMI, SBP. Model 3: Model 2 + HbA1c, CRP, TG. sHR, subdistribution hazard ratio; CI: confidence interval.

**Table A15:** Frailty transition group and incident arthritis risk in Fine–Gray competing-risk analysis.

Frailty Transition	Events/*n*	Model 1			Model 2			Model 3		
		sHR	95% CI	*p*	sHR	95% CI	*p*	sHR	95% CI	*p*
Stable robust	521/3018	1.00 (reference)	–	–	1.00 (reference)	–	–	1.00 (reference)	–	–
Robust to pre-frail/frail	170/662	1.56	1.31–1.85	<0.001	1.67	1.38–2.02	<0.001	1.65	1.33–2.05	<0.001
Stable pre-frail	179/581	1.00 (reference)	–	–	1.00 (reference)	–	–	1.00 (reference)	–	–
Pre-frail to robust	119/437	0.86	0.69–1.08	0.200	0.85	0.67–1.09	0.190	0.71	0.54–0.95	0.022
Pre-frail to frail	44/129	1.20	0.87–1.65	0.270	1.10	0.76–1.59	0.610	1.10	0.73–1.66	0.650
Stable frail	28/64	1.00 (reference)	–	–	1.00 (reference)	–	–	1.00 (reference)	–	–
Frail to robust/pre-frail	32/91	0.84	0.51–1.39	0.500	0.51	0.28–0.92	0.026	0.46	0.23–0.90	0.024

Competing event: mortality. Model 1: age, sex. Model 2: Model 1 + education, marital status, smoking, drinking, physical activity, BMI, SBP. Model 3: Model 2 + HbA1c, CRP, TG. sHR, subdistribution hazard ratio; CI: confidence interval.

**Table A16:** Frailty status across two assessments and incident arthritis risk in Fine–Gray competing-risk analysis.

Frailty Status of Two Survey	Events/*n*	Model 1			Model 2			Model 3		
		sHR	95% CI	*p*	sHR	95% CI	*p*	sHR	95% CI	*p*
Frail or pre-frail	572/1964	1.00 (reference)	–	–	1.00 (reference)	–	–	1.00 (reference)	–	–
Stable robust	521/3018	0.56	0.50–0.63	<0.001	0.54	0.47–0.61	<0.001	0.54	0.47–0.63	<0.001

Competing event: mortality. Model 1: age, sex. Model 2: Model 1 + education, marital status, smoking, drinking, physical activity, BMI, SBP. Model 3: Model 2 + HbA1c, CRP, TG. sHR, subdistribution hazard ratio; CI: confidence interval.

**Table A17:** Total FI and incident arthritis risk in Fine–Gray competing-risk analysis.

Term	Events/*n*	Model 1			Model 2			Model 3		
		sHR	95% CI	*p*	sHR	95% CI	*p*	sHR	95% CI	*p*
T1 of total FI	249/1649	1.00 (reference)	–	–	1.00 (reference)	–	–	1.00 (reference)	–	–
T2 of total FI	358/1643	1.50	1.27–1.76	<0.001	1.60	1.33–1.91	<0.001	1.53	1.24–1.89	<0.001
T3 of total FI	486/1690	1.96	1.68–2.30	<0.001	2.18	1.83–2.59	<0.001	2.1	1.71–2.57	<0.001
*p* for trend		<0.001			<0.001			<0.001		

Competing event: mortality. Model 1: age, sex. Model 2: Model 1 + education, marital status, smoking, drinking, physical activity, BMI, SBP. Model 3: Model 2 + HbA1c, CRP, TG. sHR, subdistribution hazard ratio; CI: confidence interval.

**Table A18:** ΔFI and incident arthritis risk in Fine–Gray competing-risk analysis.

Term	Events/*n*	Model 1			Model 2			Model 3		
		sHR	95% CI	*p*	sHR	95% CI	*p*	sHR	95% CI	*p*
T1 of ΔFI	397/1692	1.00 (reference)	–	–	1.00 (reference)	–	–	1.00 (reference)	–	–
T2 of ΔFI	262/1596	0.69	0.59–0.80	<0.001	0.73	0.62–0.86	<0.001	0.73	0.60–0.89	0.002
T3 of ΔFI	434/1694	1.08	0.95–1.24	0.240	1.18	1.02–1.37	0.025	1.26	1.07–1.49	0.006
*p* for trend		0.250			0.032			0.009		

Competing event: mortality. Model 1: age, sex. Model 2: Model 1 + education, marital status, smoking, drinking, physical activity, BMI, SBP. Model 3: Model 2 + HbA1c, CRP, TG. sHR, subdistribution hazard ratio; CI: confidence interval.

**Table A19:** Baseline frailty category and incident arthritis risk stratified by age group.

Frailty Status	Events/*n*	Model 1			Model 2			Model 3		
		HR	95% CI	*p*	HR	95% CI	*p*	HR	95% CI	*p*
Age < 60 years										
Robust	462/2500	1.00 (reference)	–	–	1.00 (reference)	–	–	1.00 (reference)	–	–
Pre-frail	197/641	1.79	1.52–2.12	<0.001	1.76	1.46–2.12	<0.001	1.75	1.42–2.17	<0.001
Frail	26/74	1.46	1.20–1.78	<0.001	1.60	1.29–2.00	<0.001	1.55	1.22–1.97	<0.001
Age ≥ 60 years										
Robust	229/1180	1.00 (reference)	–	–	1.00 (reference)	–	–	1.00 (reference)	–	–
Pre-frail	145/506	1.55	1.26–1.91	<0.001	1.65	1.30–2.08	<0.001	1.54	1.18–2.02	0.002
Frail	34/81	1.58	1.32–1.89	<0.001	1.77	1.44–2.18	<0.001	1.79	1.41–2.28	<0.001
*p* for interaction		0.707			0.939			0.904		

Model 1: sex. Model 2: Model 1 + education, marital status, smoking, drinking, physical activity, BMI, SBP. Model 3: Model 2 + HbA1c, CRP, TG. HR, hazard ratio; CI: confidence interval.

**Table A20:** Frailty transition group and incident arthritis risk stratified by age group.

Frailty Transition	Events/*n*	Model 1			Model 2			Model 3		
		HR	95% CI	*p*	HR	95% CI	*p*	HR	95% CI	*p*
Age < 60 years										
Stable robust	359/2124	1.00 (reference)	–	–	1.00 (reference)	–	–	1.00 (reference)	–	–
Robust to pre-frail/frail	103/376	1.70	1.36–2.12	<0.001	1.82	1.43–2.31	<0.001	1.75	1.32–2.32	<0.001
Age ≥ 60 years										
Stable robust	162/894	1.00 (reference)	–	–	1.00 (reference)	–	–	1.00 (reference)	–	–
Robust to pre-frail/frail	67/286	1.37	1.03–1.82	0.032	1.51	1.09–2.08	0.013	1.58	1.09–2.29	0.017
*p* for interaction			0.198			0.224			0.575	
Age < 60 years										
Stable pre-frail	105/318	1.00 (reference)	–	–	1.00 (reference)	–	–	1.00 (reference)	–	–
Pre-frail to robust	73/262	0.81	0.60–1.09	0.160	0.82	0.59–1.13	0.226	0.69	0.47–1.01	0.057
Pre-frail to frail	19/61	0.98	0.60–1.60	0.945	0.77	0.42–1.43	0.409	0.80	0.40–1.59	0.526
Age ≥ 60 years										
Stable pre-frail	74/263	1.00 (reference)	–	–	1.00 (reference)	–	–	1.00 (reference)	–	–
Pre-frail to robust	46/175	0.95	0.65–1.37	0.768	0.90	0.60–1.36	0.628	0.74	0.45–1.21	0.226
Pre-frail to frail	25/68	1.58	1.01–2.49	0.049	1.69	1.00–2.85	0.050	1.65	0.92–2.96	0.093
*p* for interaction			0.159			0.059			0.086	
Age < 60 years										
Stable frail	10/24	1.00 (reference)	–	–	1.00 (reference)	–	–	1.00 (reference)	–	–
Frail to robust/pre-frail	16/50	0.68	0.30–1.51	0.341	0.63	0.23–1.70	0.361	0.02	0.00–0.21	<0.001
Age ≥ 60 years										
Stable frail	18/40	1.00 (reference)	–	–	1.00 (reference)	–	–	1.00 (reference)	–	–
Frail to robust/pre-frail	16/41	0.85	0.43–1.68	0.640	0.33	0.13–0.85	0.022	0.37	0.12–1.15	0.087
*p* for interaction		0.746			0.927			0.517		

Model 1: sex. Model 2: Model 1 + education, marital status, smoking, drinking, physical activity, BMI, SBP. Model 3: Model 2 + HbA1c, CRP, TG. HR, hazard ratio; CI: confidence interval.

**Table A21:** Frailty status across two assessments and incident arthritis risk stratified by age group.

Frailty Status of Two Survey	Events/*n*	Model 1			Model 2			Model 3		
		HR	95% CI	*p*	HR	95% CI	*p*	HR	95% CI	*p*
Age < 60 years										
Frail or pre-frail	326/1091	1.00 (reference)	–	–	1.00 (reference)	–	–	1.00 (reference)	–	–
Stable robust	359/2124	0.52	0.45–0.61	<0.001	0.50	0.43–0.60	<0.001	0.51	0.42–0.62	<0.001
Age ≥ 60 years										
Frail or pre-frail	246/873	1.00 (reference)	–	–	1.00 (reference)	–	–	1.00 (reference)	–	–
Stable robust	162/894	0.61	0.50–0.75	<0.001	0.56	0.45–0.70	<0.001	0.57	0.44–0.73	<0.001
*p* for interaction		0.302			0.406			0.604		

Model 1: sex. Model 2: Model 1 + education, marital status, smoking, drinking, physical activity, BMI, SBP. Model 3: Model 2 + HbA1c, CRP, TG. HR, hazard ratio; CI: confidence interval.

**Table A22:** Total FI and incident arthritis risk stratified by age group.

Term	Events/*n*	Model 1			Model 2			Model 3		
		HR	95% CI	*p*	HR	95% CI	*p*	HR	95% CI	*p*
Age < 60 years										
T1 of total FI	173/1234	1.00 (reference)	–	–	1.00 (reference)	–	–	1.00 (reference)	–	–
T2 of total FI	242/1060	1.70	1.40–2.07	<0.001	1.79	1.44–2.22	<0.001	1.70	1.31–2.19	<0.001
T3 of total FI	270/921	1.51	1.37–1.67	<0.001	1.58	1.42–1.76	<0.001	1.55	1.36–1.76	<0.001
*p* for trend		<0.001			<0.001			<0.001		
Age ≥ 60 years										
T1 of total FI	76/415	1.00 (reference)	–	–	1.00 (reference)	–	–	1.00 (reference)	–	–
T2 of total FI	116/583	1.10	0.82–1.46	0.538	1.28	0.93–1.77	0.127	1.26	0.86–1.85	0.231
T3 of total FI	216/769	1.25	1.10–1.43	<0.001	1.37	1.18–1.59	<0.001	1.35	1.14–1.61	<0.001
*p* for trend		<0.001			<0.001			<0.001		
*p* for interaction		0.120			0.243			0.483		

Model 1: sex. Model 2: Model 1 + education, marital status, smoking, drinking, physical activity, BMI, SBP. Model 3: Model 2 + HbA1c, CRP, TG. HR, hazard ratio; CI: confidence interval.

**Table A23:** ΔFI and incident arthritis risk stratified by age group.

Term	Events/*n*	Model 1			Model 2			Model 3		
		HR	95% CI	*p*	HR	95% CI	*p*	HR	95% CI	*p*
Age < 60 years										
T1 of ΔFI	244/1094	1.00 (reference)	–	–	1.00 (reference)	–	–	1.00 (reference)	–	–
T2 of ΔFI	172/1093	0.68	0.56–0.83	<0.001	0.73	0.58–0.90	0.004	0.77	0.60–0.99	0.047
T3 of ΔFI	269/1028	1.09	1.00–1.18	0.062	1.15	1.05–1.27	0.004	1.18	1.06–1.32	0.003
*p* for trend		0.058			0.003			0.003		
Age ≥ 60 years										
T1 of ΔFI	153/598	1.00 (reference)	–	–	1.00 (reference)	–	–	1.00 (reference)	–	–
T2 of ΔFI	90/503	0.68	0.52–0.88	0.004	0.7	0.53–0.94	0.016	0.64	0.45–0.90	0.010
T3 of ΔFI	165/666	0.98	0.88–1.09	0.72	0.99	0.88–1.12	0.924	1.05	0.91–1.20	0.531
*p* for trend		0.768			0.929			0.498		
*p* for interaction		0.168			0.047			0.197		

Model 1: sex. Model 2: Model 1 + education, marital status, smoking, drinking, physical activity, BMI, SBP. Model 3: Model 2 + HbA1c, CRP, TG. HR, hazard ratio; CI: confidence interval.

**Table A24:** Baseline frailty category and incident arthritis risk stratified by sex.

Frailty Status	Events/*n*	Model 1			Model 2			Model 3		
		HR	95% CI	*p*	HR	95% CI	*p*	HR	95% CI	*p*
Males										
Robust	377/2107	1.00 (reference)	–	–	1.00 (reference)	–	–	1.00 (reference)	–	–
Pre-frail	134/534	1.51	1.24–1.84	<0.001	1.49	1.19–1.86	<0.001	1.46	1.12–1.89	0.005
Frail	25/70	1.49	1.21–1.82	<0.001	1.62	1.30–2.03	<0.001	1.74	1.36–2.21	<0.001
Females										
Robust	314/1573	1.00 (reference)	–	–	1.00 (reference)	–	–	1.00 (reference)	–	–
Pre-frail	208/613	1.89	1.58–2.26	<0.001	1.93	1.59–2.34	<0.001	1.87	1.50–2.33	<0.001
Frail	35/85	1.58	1.33–1.89	<0.001	1.77	1.44–2.17	<0.001	1.57	1.23–1.99	<0.001
*p* for interaction		0.164			0.287			0.790		

Model 1: age. Model 2: Model 1 + education, marital status, smoking, drinking, physical activity, BMI, SBP. Model 3: Model 2 + HbA1c, CRP, TG. HR, hazard ratio; CI: confidence interval.

**Table A25:** Frailty transition group and incident arthritis risk stratified by sex.

Frailty Transition	Events/*n*	Model 1			Model 2			Model 3		
		HR	95% CI	*p*	HR	95% CI	*p*	HR	95% CI	*p*
Males										
Stable robust	296/1792	1.00 (reference)	–	–	1.00 (reference)	–	–	1.00 (reference)	–	–
Robust to pre-frail/frail	81/315	1.66	1.29–2.13	<0.001	1.71	1.29–2.25	<0.001	1.66	1.20–2.30	0.002
Females										
Stable robust	225/1226	1.00 (reference)	–	–	1.00 (reference)	–	–	1.00 (reference)	–	–
Robust to pre-frail/frail	89/347	1.52	1.19–1.94	<0.001	1.66	1.27–2.17	<0.001	1.66	1.22–2.27	0.001
*p* for interaction			0.499			0.680			0.808	
Males										
Stable pre-frail	59/256	1.00 (reference)	–	–	1.00 (reference)	–	–	1.00 (reference)	–	–
Pre-frail to robust	56/209	1.13	0.78–1.64	0.506	1.02	0.67–1.53	0.941	0.80	0.48–1.32	0.380
Pre-frail to frail	19/69	1.34	0.80–2.25	0.266	1.18	0.59–2.34	0.643	1.68	0.78–3.59	0.183
Females										
Stable pre-frail	120/325	1.00 (reference)	–	–	1.00 (reference)	–	–	1.00 (reference)	–	–
Pre-frail to robust	63/228	0.72	0.53–0.97	0.032	0.72	0.52–1.01	0.053	0.61	0.42–0.90	0.013
Pre-frail to frail	25/60	1.18	0.76–1.82	0.461	1.09	0.68–1.76	0.721	0.96	0.56–1.66	0.889
*p* for interaction			0.230			0.531			0.329	
Males										
Stable frail	11/29	1.00 (reference)	–	–	1.00 (reference)	–	–	1.00 (reference)	–	–
Frail to robust/pre-frail	14/41	0.84	0.37–1.92	0.685	0.38	0.12–1.20	0.101	0.26	0.06–1.20	0.09
Females										
Stable frail	17/35	1.00 (reference)	–	–	1.00 (reference)	–	–	1.00 (reference)	–	–
Frail to robust/pre-frail	18/50	0.81	0.40–1.61	0.539	0.33	0.12–0.91	0.032	0.14	0.03–0.55	0.005
*p* for interaction		0.734			0.257			0.031		

Model 1: age. Model 2: Model 1 + education, marital status, smoking, drinking, physical activity, BMI, SBP. Model 3: Model 2 + HbA1c, CRP, TG. HR, hazard ratio; CI: confidence interval.

**Table A26:** Frailty status across two assessments and incident arthritis risk stratified by sex.

Frailty Status of Two Survey	Events/*n*	Model 1			Model 2			Model 3		
		HR	95% CI	*p*	HR	95% CI	*p*	HR	95% CI	*p*
Males										
Frail or pre-frail	240/919	1.00 (reference)	–	–	1.00 (reference)	–	–	1.00 (reference)	–	–
Stable robust	296/1792	0.57	0.48–0.68	<0.001	0.56	0.46–0.68	<0.001	0.56	0.45–0.70	<0.001
Females										
Frail or pre-frail	332/1045	1.00 (reference)	–	–	1.00 (reference)	–	–	1.00 (reference)	–	–
Stable robust	225/1226	0.53	0.44–0.62	<0.001	0.49	0.41–0.59	<0.001	0.50	0.40–0.62	<0.001
*p* for interaction		0.301			0.362			0.496		

Model 1: age. Model 2: Model 1 + education, marital status, smoking, drinking, physical activity, BMI, SBP. Model 3: Model 2 + HbA1c, CRP, TG. HR, hazard ratio; CI: confidence interval.

**Table A27:** Total FI and incident arthritis risk stratified by sex.

Term	Events/*n*	Model 1			Model 2			Model 3		
		HR	95% CI	*p*	HR	95% CI	*p*	HR	95% CI	*p*
Males										
T1 of total FI	149/1041	1.00 (reference)	–	–	1.00 (reference)	–	–	1.00 (reference)	–	–
T2 of total FI	189/893	1.56	1.25–1.93	<0.001	1.51	1.19–1.93	<0.001	1.45	1.09–1.93	0.01
T3 of total FI	198/777	1.42	1.27–1.59	<0.001	1.47	1.30–1.67	<0.001	1.47	1.27–1.70	<0.001
*p* for trend		<0.001			<0.001			<0.001		
Females										
T1 of total FI	100/608	1.00 (reference)	–	–	1.00 (reference)	–	–	1.00 (reference)	–	–
T2 of total FI	169/750	1.46	1.13–1.87	0.003	1.72	1.31–2.26	<0.001	1.64	1.19–2.26	0.002
T3 of total FI	288/913	1.43	1.28–1.61	<0.001	1.54	1.35–1.75	<0.001	1.50	1.29–1.74	<0.001
*p* for trend		<0.001			<0.001			<0.001		
*p* for interaction		0.380			0.562			0.560		

Model 1: age. Model 2: Model 1 + education, marital status, smoking, drinking, physical activity, BMI, SBP. Model 3: Model 2 + HbA1c, CRP, TG. HR, hazard ratio; CI: confidence interval.

**Table A28:** ΔFI and incident arthritis risk stratified by sex.

Term	Events/*n*	Model 1			Model 2			Model 3		
		HR	95% CI	*p*	HR	95% CI	*p*	HR	95% CI	*p*
Males										
T1 of ΔFI	192/906	1.00 (reference)	–	–	1.00 (reference)	–	–	1.00 (reference)	–	–
T2 of ΔFI	150/969	0.70	0.57–0.87	0.001	0.76	0.60–0.97	0.025	0.73	0.55–0.96	0.025
T3 of ΔFI	194/836	1.06	0.96–1.17	0.258	1.09	0.98–1.22	0.115	1.11	0.98–1.27	0.098
*p* for trend		0.303			0.144			0.109		
Females										
T1 of ΔFI	205/786	1.00 (reference)	–	–	1.00 (reference)	–	–	1.00 (reference)	–	–
T2 of ΔFI	112/627	0.66	0.52–0.83	<0.001	0.68	0.52–0.87	0.003	0.71	0.53–0.96	0.025
T3 of ΔFI	240/858	1.03	0.94–1.13	0.488	1.09	0.99–1.21	0.091	1.14	1.01–1.28	0.028
*p* for trend		0.426			0.076			0.021		
*p* for interaction		0.914			0.973			0.752		

Model 1: age. Model 2: Model 1 + education, marital status, smoking, drinking, physical activity, BMI, SBP. Model 3: Model 2 + HbA1c, CRP, TG. HR, hazard ratio; CI: confidence interval.

**Table A29:** Baseline frailty category and incident arthritis risk with a wave-based event definition.

Frailty Status	Events/*n*	Model 1			Model 2			Model 3		
		HR	95% CI	*p*	HR	95% CI	*p*	HR	95% CI	*p*
Robust	691/3680	1.00 (reference)	–	–	1.00 (reference)	–	–	1.00 (reference)	–	–
Pre-frail	342/1147	1.70	1.49–1.94	<0.001	1.71	1.47–1.97	<0.001	1.65	1.40–1.95	<0.001
Frail	60/155	2.30	1.76–3.00	<0.001	2.74	2.03–3.69	<0.001	2.61	1.87–3.65	<0.001

In the wave-based sensitivity analysis, incident arthritis was assigned to the survey wave when it was first reported. Model 1: age. Model 2: Model 1 + education, marital status, smoking, drinking, physical activity, BMI, SBP. Model 3: Model 2 + HbA1c, CRP, TG. HR, hazard ratio; CI: confidence interval.

**Table A30:** Frailty transition group and incident arthritis risk with a wave-based event definition.

Frailty Transition	Events/*n*	Model 1			Model 2			Model 3		
		HR	95% CI	*p*	HR	95% CI	*p*	HR	95% CI	*p*
Stable robust	521/3018	1.00 (reference)	–	–	1.00 (reference)	–	–	1.00 (reference)	–	–
Robust to pre-frail/frail	170/662	1.61	1.35–1.93	<0.001	1.69	1.40–2.05	<0.001	1.67	1.34–2.09	<0.001
Stable pre-frail	179/581	1.00 (reference)	–	–	1.00 (reference)	–	–	1.00 (reference)	–	–
Pre-frail to robust	119/437	0.88	0.69–1.11	0.268	0.86	0.66–1.10	0.230	0.72	0.53–0.96	0.028
Pre-frail to frail	44/129	1.25	0.90–1.75	0.182	1.15	0.78–1.69	0.489	1.19	0.78–1.82	0.431
Stable frail	28/64	1.00 (reference)	–	–	1.00 (reference)	–	–	1.00 (reference)	–	–
Frail to robust/pre-frail	32/91	0.84	0.49–1.41	0.502	0.48	0.25–0.90	0.023	0.45	0.21–0.93	0.030

In the wave-based sensitivity analysis, incident arthritis was assigned to the survey wave when it was first reported. Model 1: age. Model 2: Model 1 + education, marital status, smoking, drinking, physical activity, BMI, SBP. Model 3: Model 2 + HbA1c, CRP, TG. HR, hazard ratio; CI: confidence interval.

**Table A31:** Frailty status across two assessments and incident arthritis risk with a wave-based event definition.

Frailty Status of Two Survey	Events/*n*	Model 1			Model 2			Model 3		
		HR	95% CI	*p*	HR	95% CI	*p*	HR	95% CI	*p*
Frail or pre-frail	572/1964	1.00 (reference)	–	–	1.00 (reference)	–	–	1.00 (reference)	–	–
Stable robust	521/3018	0.55	0.48–0.62	<0.001	0.53	0.46–0.60	<0.001	0.54	0.46–0.63	<0.001

In the wave-based sensitivity analysis, incident arthritis was assigned to the survey wave when it was first reported. Model 1: age. Model 2: Model 1 + education, marital status, smoking, drinking, physical activity, BMI, SBP. Model 3: Model 2 + HbA1c, CRP, TG. HR, hazard ratio; CI: confidence interval.

**Table A32:** Total FI and incident arthritis risk with a wave-based event definition.

Term	Events/*n*	Model 1			Model 2			Model 3		
		HR	95% CI	*p*	HR	95% CI	*p*	HR	95% CI	*p*
T1 of total FI	249/1649	1.00 (reference)	–	–	1.00 (reference)	–	–	1.00 (reference)	–	–
T2 of total FI	358/1643	1.49	1.27–1.76	<0.001	1.58	1.32–1.89	<0.001	1.54	1.25–1.89	<0.001
T3 of total FI	486/1690	2.09	1.78–2.44	<0.001	2.31	1.94–2.75	<0.001	2.18	1.78–2.67	<0.001
*p* for trend		<0.001			<0.001			<0.001		

In the wave-based sensitivity analysis, incident arthritis was assigned to the survey wave when it was first reported. Model 1: age. Model 2: Model 1 + education, marital status, smoking, drinking, physical activity, BMI, SBP. Model 3: Model 2 + HbA1c, CRP, TG. HR, hazard ratio; CI: confidence interval.

**Table A33:** ΔFI and incident arthritis risk with a wave-based event definition.

Term	Events/*n*	Model 1			Model 2			Model 3		
		HR	95% CI	*p*	HR	95% CI	*p*	HR	95% CI	*p*
T1 of ΔFI	397/1692	1.00 (reference)	–	–	1.00 (reference)	–	–	1.00 (reference)	–	–
T2 of ΔFI	262/1596	0.67	0.58–0.79	<0.001	0.71	0.60–0.85	<0.001	0.71	0.58–0.87	0.001
T3 of ΔFI	434/1694	1.10	0.96–1.26	0.188	1.21	1.04–1.41	0.011	1.29	1.09–1.53	0.004
*p* for trend		0.575			0.198			0.018		

In the wave-based sensitivity analysis, incident arthritis was assigned to the survey wave when it was first reported. Model 1: age. Model 2: Model 1 + education, marital status, smoking, drinking, physical activity, BMI, SBP. Model 3: Model 2 + HbA1c, CRP, TG. HR, hazard ratio; CI: confidence interval.

**Table A34:** Baseline frailty category and incident arthritis risk with a 27-item Frailty Index.

Frailty Status	Events/*n*	Model 1			Model 2			Model 3		
		HR	95% CI	*p*	HR	95% CI	*p*	HR	95% CI	*p*
Robust	829/4143	1.00 (reference)	–	–	1.00 (reference)	–	–	1.00 (reference)	–	–
Pre-frail	259/860	1.60	1.39–1.84	<0.001	1.68	1.43–1.96	<0.001	1.54	1.28–1.85	<0.001
Frail	32/78	2.26	1.59–3.22	<0.001	2.53	1.65–3.87	<0.001	2.28	1.40–3.70	<0.001

In this sensitivity analysis, a 27-item Frailty Index (27-item FI) was constructed after excluding three mobility-related items (difficulty climbing stairs, stooping/kneeling/crouching, and reaching arms overhead). Model 1: age. Model 2: Model 1 + education, marital status, smoking, drinking, physical activity, BMI, SBP. Model 3: Model 2 + HbA1c, CRP, TG. HR, hazard ratio; CI: confidence interval.

**Table A35:** Frailty transition group and incident arthritis risk with a 27-item Frailty Index.

Frailty Transition	Events/*n*	Model 1			Model 2			Model 3		
		HR	95% CI	*p*	HR	95% CI	*p*	HR	95% CI	*p*
Stable robust	681/3603	1.00 (reference)	–	–	1.00 (reference)	–	–	1.00 (reference)	–	–
Robust to pre-frail/frail	148/540	1.55	1.29–1.85	<0.001	1.61	1.32–1.97	<0.001	1.63	1.31–2.05	<0.001
Stable pre-frail	145/433	1.00 (reference)	–	–	1.00 (reference)	–	–	1.00 (reference)	–	–
Pre-frail to robust	94/360	0.74	0.57–0.96	0.023	0.74	0.55–0.99	0.046	0.69	0.48–0.98	0.038
Pre-frail to frail	20/67	0.93	0.58–1.49	0.762	1.04	0.60–1.79	0.890	1.28	0.72–2.28	0.405
Stable frail	14/31	1.00 (reference)	–	–	1.00 (reference)	–	–	1.00 (reference)	–	–
Frail to robust/pre-frail	18/47	1.04	0.49–2.19	0.19	0.28	0.09–0.92	0.036	0.31	0.09–1.14	0.077

In this sensitivity analysis, a 27-item Frailty Index (27-item FI) was constructed after excluding three mobility-related items (difficulty climbing stairs, stooping/kneeling/crouching, and reaching arms overhead). Model 1: age. Model 2: Model 1 + education, marital status, smoking, drinking, physical activity, BMI, SBP. Model 3: Model 2 + HbA1c, CRP, TG. HR, hazard ratio; CI: confidence interval.

**Table A36:** Frailty status across two assessments and incident arthritis risk with a 27-item Frailty Index.

Frailty Status of Two Survey	Events/*n*	Model 1			Model 2			Model 3		
		HR	95% CI	*p*	HR	95% CI	*p*	HR	95% CI	*p*
Frail or pre-frail	439/1478	1.00 (reference)	–	–	1.00 (reference)	–	–	1.00 (reference)	–	–
Stable robust	681/3603	0.60	0.53–0.67	<0.001	0.57	0.50–0.65	<0.001	0.59	0.51–0.70	<0.001

In this sensitivity analysis, a 27-item Frailty Index (27-item FI) was constructed after excluding three mobility-related items (difficulty climbing stairs, stooping/kneeling/crouching, and reaching arms overhead). Model 1: age. Model 2: Model 1 + education, marital status, smoking, drinking, physical activity, BMI, SBP. Model 3: Model 2 + HbA1c, CRP, TG. HR, hazard ratio; CI: confidence interval.

**Table A37:** Total FI and incident arthritis risk with a 27-item Frailty Index.

Term	Events/*n*	Model 1			Model 2			Model 3		
		HR	95% CI	*p*	HR	95% CI	*p*	HR	95% CI	*p*
T1 of total FI	259/1677	1.00 (reference)	–	–	1.00 (reference)	–	–	1.00 (reference)	–	–
T2 of total FI	385/1677	1.55	1.32–1.81	<0.001	1.69	1.42–2.01	<0.001	1.78	1.45–2.19	<0.001
T3 of total FI	476/1727	1.92	1.65–2.24	<0.001	2.22	1.87–2.64	<0.001	2.29	1.87–2.81	<0.001
*p* for trend		<0.001			<0.001			<0.001		

In this sensitivity analysis, a 27-item Frailty Index (27-item FI) was constructed after excluding three mobility-related items (difficulty climbing stairs, stooping/kneeling/crouching, and reaching arms overhead). Model 1: age. Model 2: Model 1 + education, marital status, smoking, drinking, physical activity, BMI, SBP. Model 3: Model 2 + HbA1c, CRP, TG. HR, hazard ratio; CI: confidence interval.

**Table A38:** ΔFI and incident arthritis risk with a 27-item Frailty Index.

Term	Events/*n*	Model 1			Model 2			Model 3		
		HR	95% CI	*p*	HR	95% CI	*p*	HR	95% CI	*p*
T1 of ΔFI	392/1684	1.00 (reference)	–	–	1.00 (reference)	–	–	1.00 (reference)	–	–
T2 of ΔFI	277/1674	0.69	0.59–0.80	<0.001	0.75	0.63–0.89	<0.001	0.76	0.62–0.93	0.007
T3 of ΔFI	451/1723	1.13	0.99–1.30	0.071	1.22	1.05–1.42	0.010	1.33	1.12–1.58	0.001
*p* for trend		0.239			0.052			<0.001		

In this sensitivity analysis, a 27-item Frailty Index (27-item FI) was constructed after excluding three mobility-related items (difficulty climbing stairs, stooping/kneeling/crouching, and reaching arms overhead). Model 1: age. Model 2: Model 1 + education, marital status, smoking, drinking, physical activity, BMI, SBP. Model 3: Model 2 + HbA1c, CRP, TG. HR, hazard ratio; CI: confidence interval.

**Table A39:** Baseline characteristics of the overall sample and comparisons between included and excluded participants.

Variable	Overall	Included	Excluded	*p* Value
*n*	16,931	4982	11,949	
Age, mean (SD), years	59.11 (9.8)	56.98 (8.5)	59.99 (10.1)	<0.001
Sex, *n* (%)				
Male	8268 (48.8)	2711 (54.4)	5557 (46.5)	<0.001
Female	8663 (51.2)	2271 (45.6)	6392 (53.5)	
Smoking status, *n* (%)				
Never smokers	8116 (55.9)	2672 (53.6)	5444 (57.1)	<0.001
Ever smokers	6394 (44.1)	2310 (46.4)	4084 (42.9)	
Drinking status, *n* (%)				
Never drinkers	7978 (55.1)	2536 (51.0)	5442 (57.3)	<0.001
Ever drinkers	6495 (44.9)	2437 (49.0)	4058 (42.7)	
Marital status, *n* (%)				
Other marital status	2014 (13.8)	473 (9.5)	1541 (16.0)	<0.001
Married or partnered	12,594 (86.2)	4509 (90.5)	8085 (84.0)	
Education, *n* (%)				
Lower than secondary	14,848 (87.7)	4150 (83.3)	10,698 (89.6)	<0.001
Upper secondary & vocational training	1732 (10.2)	715 (14.4)	1017 (8.5)	
Tertiary education	345 (2.0)	117 (2.3)	228 (1.9)	
Physical activity, *n* (%)				
Light	5516 (42.4)	1682 (39.3)	3834 (43.9)	<0.001
Moderate	3653 (28.1)	1321 (30.9)	2332 (26.7)	
Vigorous	3843 (29.5)	1272 (29.8)	2571 (29.4)	
BMI, median (IQR), kg/m^2^	23.08 (20.8, 25.7)	23.32 (21.0, 25.8)	22.94 (20.6, 25.6)	<0.001
SBP, mean (SD), mmHg	129.86 (21.5)	127.95 (20.2)	130.78 (22.1)	<0.001
HbA1c, mean (SD), %	5.27 (0.8)	5.25 (0.8)	5.28 (0.8)	0.052
TG, mean (SD), mg/dL	134.67 (109.0)	133.49 (103.1)	135.22 (111.7)	0.432
CRP, median (IQR), mg/L	1.05 (0.6, 2.2)	1.02 (0.5, 2.1)	1.06 (0.6, 2.3)	0.004

Group differences were assessed with the Kruskal–Wallis H test (continuous variables) and the chi-square test (categorical variables). BMI, body mass index; SBP, systolic blood pressure; HbA1c, glycated hemoglobin; TG, triglycerides; CRP, C-reactive protein; IQR, interquartile range.

**Table A40:** Comparison of variable distributions before and after multiple imputation.

Variable	Before	After
Age, mean (SD), years	58.97 (8.54)	58.97 (8.54)
Sex, *n* (%)		
Male	2711 (54.42)	2711 (54.42)
Female	2271 (45.58)	2271 (45.58)
Smoking status, *n* (%)		
Never smokers	2672 (53.63)	2672 (53.63)
Ever smokers	2310 (46.37)	2310 (46.37)
Drinking status, *n* (%)		
Never drinkers	2535 (50.98)	2536 (51.00)
Ever drinkers	2438 (49.02)	2437 (49.00)
Marital status, *n* (%)		
Other marital status	473 (9.49)	473 (9.49)
Married or partnered	4509 (90.51)	4509 (90.51)
Education, *n* (%)		
Lower than secondary	4369 (87.70)	4150 (83.30)
Upper secondary & vocational training	508 (10.20)	715 (14.35)
Tertiary education	100 (2.00)	117 (2.35)
Physical activity, *n* (%)		
Light	1685 (39.42)	1682 (39.35)
Moderate	1343 (31.41)	1321 (30.90)
Vigorous	1247 (29.16)	1272 (29.75)
BMI, median (IQR), kg/m^2^	23.63 (21.29, 26.13)	23.65 (21.30,26.17)
SBP, mean (SD), mmHg	128.75 (20.67)	128.79 (20.54)
HbA1c, mean (SD), %	5.27 (0.77)	5.25 (0.80)
TG, mean (SD), mg/dL	134.46 (105.25)	133.49 (103.07)
CRP, median (IQR), mg/L	1.05 (0.54, 2.09)	1.02 (0.54–2.08)

Continuous variables were expressed as mean (SD), median (interquartile range), and categorical variables were expressed as numbers (%). BMI, body mass index; SBP, systolic blood pressure; HbA1c, glycated hemoglobin; TG, triglycerides; CRP, C-reactive protein; IQR, interquartile range.

**Table A41:** Results of the Proportional Hazards (PHs) Assumption Test in Model 3 for the Cox Regression Analysis.

Table	Group	Chi-Square (χ^2^)	*p*-Value	PH Assumption Met (Yes/No)
Table 5	Robust individuals	0.694	0.405	Yes
	GLOBAL	9.603	0.726	
	Pre-frail individuals	0.177	0.915	Yes
	GLOBAL	9.020	0.830	
	Frail individuals	0.503	0.480	Yes
	GLOBAL	7.698	0.86	
Table 6	Frail or pre-frail	1.205	0.272	Yes
	GLOBAL	11.298	0.586	
Table 7	Total FI	3.168	0.205	Yes
	GLOBAL	13.232	0.508	
Table 8	ΔFI	1.019	0.601	Yes
	GLOBAL	10.094	0.755	

**Table A42:** Absolute risk of incident arthritis across frailty-transition categories.

Frailty Transition	N	Events	Person-Years	Incidence (per 1000 PY)
Stable robust	3018	521	13,200	39.5
Robust to pre-frail/frail	662	170	2678	63.5
Pre-frail to robust	437	119	1750	68.0
Pre-frail to frail	129	44.000	461	95.4
Stable pre-frail	581	179	2252	79.5
Frail to robust/pre-frail	91	32	341	93.9
Stable frail	64	28	221	126.6

For frailty-transition analyses, follow-up time was calculated from the assessment at which frailty transition status was established until incident arthritis, death, censoring, or the end of follow-up.

**Table A43:** Baseline frailty category and incident arthritis risk after excluding participants with early incident arthritis within 2 years after Wave 2.

Frailty Status	Events/*n*	Model 1			Model 2			Model 3		
		HR	95% CI	*p*	HR	95% CI	*p*	HR	95% CI	*p*
Robust	295/3284	1.00 (reference)	–	–	1.00 (reference)	–	–	1.00 (reference)	–	–
Pre-frail	124/929	1.52	1.23–1.88	<0.001	1.63	1.29–2.06	<0.001	1.80	1.37–2.36	<0.001
Frail	20/115	1.99	1.26–3.14	0.003	2.37	1.38–4.10	0.002	2.23	1.16–4.26	0.015

In this sensitivity analysis, participants who developed incident arthritis within 2 years after the Wave 2 assessment were excluded. Model 1: age. Model 2: Model 1 + education, marital status, smoking, drinking, physical activity, BMI, SBP. Model 3: Model 2 + HbA1c, CRP, TG. HR, hazard ratio; CI: confidence interval.

**Table A44:** Frailty transition group and incident arthritis risk after excluding participants with early incident arthritis within 2 years after Wave 2.

Frailty Transition	Events/*n*	Model 1			Model 2			Model 3		
		HR	95% CI	*p*	HR	95% CI	*p*	HR	95% CI	*p*
Stable robust	222/2719	1.00 (reference)	–	–	1.00 (reference)	–	–	1.00 (reference)	–	–
Robust to pre-frail/frail	73/565	1.66	1.26–2.17	<0.001	1.82	1.35–2.45	<0.001	1.60	1.10–2.32	0.014
Stable pre-frail	67/469	1.00 (reference)	–	–	1.00 (reference)	–	–	1.00 (reference)	–	–
Pre-frail to robust	44/362	0.85	0.58–1.24	0.393	0.77	0.51–1.17	0.219	0.65	0.40–1.05	0.075
Pre-frail to frail	13/98	1.04	0.58–1.90	0.886	0.87	0.43–1.77	0.699	0.92	0.43–1.97	0.830
Stable frail	10/46	1.00 (reference)	–	–	1.00 (reference)	–	–	1.00 (reference)	–	–
Frail to robust/pre-frail	10/69	0.76	0.31–1.92	0.569	0.20	0.05–0.80	0.023	0.23	0.03–1.60	0.138

In this sensitivity analysis, participants who developed incident arthritis within 2 years after the Wave 2 assessment were excluded. Model 1: age. Model 2: Model 1 + education, marital status, smoking, drinking, physical activity, BMI, SBP. Model 3: Model 2 + HbA1c, CRP, TG. HR, hazard ratio; CI: confidence interval.

**Table A45:** Frailty status across two assessments and incident arthritis risk after excluding participants with early incident arthritis within 2 years after Wave 2.

Frailty Status of Two Survey	Events/*n*	Model 1			Model 2			Model 3		
		HR	95% CI	*p*	HR	95% CI	*p*	HR	95% CI	*p*
Frail or pre-frail	217/1609	1.00 (reference)	–	–	1.00 (reference)	–	–	1.00 (reference)	–	–
Stable robust	222/2719	0.59	0.48–0.71	<0.001	0.53	0.43–0.66	<0.001	0.53	0.41–0.68	<0.001

In this sensitivity analysis, participants who developed incident arthritis within 2 years after the Wave 2 assessment were excluded. Model 1: age. Model 2: Model 1 + education, marital status, smoking, drinking, physical activity, BMI, SBP. Model 3: Model 2 + HbA1c, CRP, TG. HR, hazard ratio; CI: confidence interval.

**Table A46:** Total FI and incident arthritis risk after excluding participants with early incident arthritis within 2 years after Wave 2.

Term	Events/*n*	Model 1			Model 2			Model 3		
		HR	95% CI	*p*	HR	95% CI	*p*	HR	95% CI	*p*
T1 of total FI	111/1511	1.00 (reference)	–	–	1.00 (reference)	–	–	1.00 (reference)	–	–
T2 of total FI	152/1437	1.45	1.13–1.86	0.003	1.54	1.17–2.04	0.002	1.60	1.15–2.24	0.005
T3 of total FI	176/1380	1.78	1.40–2.28	<0.001	2.08	1.58–2.74	<0.001	2.11	1.52–2.93	<0.001
*p* for trend		<0.001			<0.001			<0.001		

In this sensitivity analysis, participants who developed incident arthritis within 2 years after the Wave 2 assessment were excluded. Model 1: age. Model 2: Model 1 + education, marital status, smoking, drinking, physical activity, BMI, SBP. Model 3: Model 2 + HbA1c, CRP, TG. HR, hazard ratio; CI: confidence interval.

**Table A47:** ΔFI and incident arthritis risk after excluding participants with early incident arthritis within 2 years after Wave 2.

Term	Events/*n*	Model 1			Model 2			Model 3		
		HR	95% CI	*p*	HR	95% CI	*p*	HR	95% CI	*p*
T1 of ΔFI	147/1442	1.00 (reference)	–	–	1.00 (reference)	–	–	1.00 (reference)	–	–
T2 of ΔFI	102/1436	0.68	0.53–0.89	0.004	0.72	0.54–0.96	0.025	0.68	0.48–0.95	0.025
T3 of ΔFI	190/1450	1.31	1.05–1.62	0.015	1.48	1.17–1.89	0.001	1.58	1.19–2.10	0.001
*p* for trend		0.743			0.183			0.067		

In this sensitivity analysis, participants who developed incident arthritis within 2 years after the Wave 2 assessment were excluded. Model 1: age. Model 2: Model 1 + education, marital status, smoking, drinking, physical activity, BMI, SBP. Model 3: Model 2 + HbA1c, CRP, TG. HR, hazard ratio; CI: confidence interval.

**Table A48:** Comparison of Cox regression results for incident arthritis using multiple imputation (MICE) and complete-case analysis.

Variable	MICE HR (95% CI)	Complete-Case HR (95% CI)
Age	1.01 (1.00–1.02)	1.01 (1.00–1.02)
Sex—Female	1.32 (1.09–1.58)	1.31 (1.02–1.69)
Smoking status	1.04 (0.87–1.23)	1.00 (0.79–1.27)
Drinking status	1.00 (0.87–1.16)	0.99 (0.82–1.18)
Marital status	1.10 (0.89–1.36)	1.01 (0.76–1.33)
Education—Upper secondary & vocational training	0.63 (0.51–0.77)	0.67 (0.51–0.89)
Education—Tertiary education	0.46 (0.26–0.79)	0.73 (0.36–1.48)
Physical activity—Moderate	1.13 (0.97–1.32)	1.11 (0.91–1.35)
Physical activity—Vigorous	1.53 (1.32–1.78)	1.44 (1.19–1.74)
BMI	1.00 (0.98–1.01)	1.00 (0.98–1.02)
SBP	1.00 (0.99–1.00)	1.00 (0.99–1.00)
HbA1c	1.05 (0.96–1.14)	1.08 (0.98–1.19)
TG	1.00 (1.00–1.00)	1.00 (1.00–1.00)
CRP	0.99 (0.98–1.01)	0.99 (0.98–1.01)

Multiple imputation analyses were performed using 20 imputed datasets with 10 iterations. Complete-case analyses included only participants with no missing data on all variables included in the model. Categorical variables were included as factor variables, with the lowest category used as the reference group. HR, hazard ratio; CI: confidence interval.
