# Supplementary material for: Frailty Transition and Risk of New-Onset Arthritis Among Adults Aged 45 Years and Older: A Longitudinal Analysis of CHARLS
Source: Healthcare (Basel). 2026 Apr 10;14(8):1000. doi: 10.3390/healthcare14081000 (PMC13115906; doi:10.3390/healthcare14081000)
Supplement: Supplementary file 1 [file healthcare-14-01000-s001.zip › healthcare-4115252-supplementary.pdf]

Supplemental Methods

Study designs

The China Health and Retirement Longitudinal Study (CHARLS) is a nationwide prospective cohort established in China. The baseline survey was launched in 2011 and enrolled 17,708 respondents from 28 provinces through a multistage probability sampling strategy, yielding a nationally representative sample. Although the study was mainly designed to include individuals aged 45 years and older, a small proportion of adults aged 40-44 years were also surveyed at baseline.

During the initial investigation, trained interviewers collected data from all participants through standardized face-to-face interviews. The questionnaire covered a wide range of variables, including demographic characteristics, lifestyle-related factors, and general health conditions. In addition to the interview survey, 13,978 participants completed physical and anthropometric assessments, from which measurements such as height, body weight, waist and hip circumferences, blood pressure, and handgrip strength were obtained. Moreover, blood specimens were collected from 11,847 individuals for laboratory evaluation. These assays provided measurements of several biochemical markers, including total cholesterol, high-density lipoprotein cholesterol, low-density lipoprotein cholesterol, glycated hemoglobin, fasting plasma glucose, and C-reactive protein.

Subsequent follow-up investigations were performed in 2013, 2015, and 2018, corresponding to waves 2, 3, and 4, respectively. These follow-up rounds continued to obtain questionnaire data and physical measurement data. A second round of blood collection was carried out in 2015, allowing the relevant biochemical parameters to be measured again.

Cohort construction followed a stepwise landmark approach:

1. Start with all participants in CHARLS Wave 1 (n = 16,931)
2. Exclude individuals with missing data required to compute baseline FI (n = 5249).
3. Exclude participants with arthritis at baseline or missing follow-up
4. For transition analysis, restrict to participants with FI data at Wave 2
5. Exclude participants reporting arthritis at Wave 2 (including those with onset between waves)
6. The final cohort (n = 4982) was defined at the Wave 2 landmark and followed prospectively for incident arthritis

Ascertainment of arthritis

In the CHARLS, arthritis was ascertained based on the self-reported physician-diagnosed arthritis. In each wave of the cohort, participants were asked "Have you been told by a doctor that you have been diagnosed with arthritis?" Those who reported being diagnosed with arthritis were considered to have arthritis. In the next wave, participants were required to confirm the existence of arthritis if they reported those in the last wave. If participants disputed self-reported arthritis from previous waves, they were corrected retrospectively. Our arthritis ascertainment was consistent with previous studies using the CHARLS cohorts[1-3].

Multiple imputation

The missing data of covariates were imputed using the multiple imputation with chained equation. Missing data were handled using multiple imputation by chained equations (MICE). A total of 20 imputed datasets were generated with 10 iterations each. The imputation model included all variables used in the analysis, as well as the outcome and follow-up time, although outcome variables were not imputed. Continuous variables were imputed using predictive mean or and median matching, while categorical variables were imputed using logistic or multinomial regression models. All variables were analyzed in their original scale without transformation. Effect estimates were computed separately for each of the 20 datasets, and then combined according to Rubin's rules. The multiple imputation was conducted using the R package "mice".

References

[1] Hootman JM, Helmick CG, Barbour KE, Theis KA, Boring MA. Updated Projected Prevalence of Self-Reported Doctor-Diagnosed Arthritis and Arthritis-Attributable Activity Limitation Among US Adults, 2015-2040. Arthritis Rheumatol. 2016;68(7):1582-1587. doi:10.1002/art.39692

[2] Yang Y, Li Y, Shui R, Li D. Association between relative fat mass and risk of arthritis: a study based on populations in China and the United States. Front Nutr. 2025;12:1555135. Published 2025 Sep 2. doi:10.3389/fnut.2025.1555135

[3] Ke WK, Xu LL, Luo N. Predictive value of insulin resistance metabolic score for cardiovascular disease in Chinese arthritis patients: a prospective cohort study. Rheumatology (Oxford). 2025;64(6):3388-3395. doi:10.1093/rheumatology/keaf048
